# *Iphiona mucronata* (Forssk.) Asch. & Schweinf. A Comprehensive Phytochemical Study via UPLC-Q-TOF-MS in the Context of the Embryo- and Cytotoxicity Profiles

**DOI:** 10.3390/molecules27217529

**Published:** 2022-11-03

**Authors:** Łukasz Pecio, Asmaa M. Otify, Fatema R. Saber, Yasser A. El-Amier, Moataz Essam Shalaby, Solomiia Kozachok, Amira K. Elmotayam, Łukasz Świątek, Adrianna Skiba, Krystyna Skalicka-Woźniak

**Affiliations:** 1 Department of Natural Products Chemistry, Medical University of Lublin, 20-093 Lublin, Poland; 2 Department of Biochemistry and Crop Quality, Institute of Soil Science and Plant Cultivation—State Research Institute, Czartoryskich 8, 24-100 Puławy, Poland; 3 Pharmacognosy Department, Faculty of Pharmacy, Cairo University, Kasr El-Aini Street, Cairo 11562, Egypt; 4 Department of Botany, Faculty of Science, Mansoura University, Mansoura 35516, Egypt; 5 Pharmaceutical Chemistry Department, Faculty of Pharmacy, Cairo University, Kasr El-Aini Street, Cairo 11562, Egypt; 6 Department of Virology with SARS Laboratory, Medical University of Lublin, 20-093 Lublin, Poland

**Keywords:** *Iphiona mucronata* (Forssk.) Asch. & Schweinf., Asteraceae, UPLC-Q-TOF-MS, sesquiterpenes, phenolics, embryotoxicity, zebrafish model, cytotoxicity

## Abstract

*Iphiona mucronata* (Family Asteraceae) is widely distributed in the Eastern desert of Egypt. It is a promising plant material for phytochemical analysis and pharmacologic studies, and so far, its specific metabolites and biological activity have not yet been thoroughly investigated. Herein, we report on the detailed phytochemical study using UPLC-Q-TOF-MS approach. This analysis allowed the putative annotation of 48 metabolites belonging to various phytochemical classes, including mostly sesquiterpenes, flavonoids, and phenolic acids. Further, zebrafish embryotoxicity has been carried out, where 100 µg/mL extract incubated for 72 h resulted in a slow touch response of the 10 examined larvae, which might be taken as a sign of a disturbed peripheral nervous system. Results of in vitro testing indicate moderate cytotoxicity towards VERO, FaDu, and HeLa cells with CC_50_ values between 91.6 and 101.7 µg/mL. However, selective antineoplastic activity in RKO cells with CC_50_ of 54.5 µg/mL was observed. To the best of our knowledge, this is the first comprehensive profile of *I. mucronata* secondary metabolites that provides chemical-based evidence for its biological effects. A further investigation should be carried out to precisely define the underlying mechanisms of toxicity.

## 1. Introduction

Tribe *Inuleae,* from the family Asteraceae, comprises a wide variety of plants with diverse ethnomedicinal uses. One good example is the genus *Inula* L., where several members of this genus have been used for various diseases and health conditions. Some of *Inula* spp. distributed in China are used as expectorants, antitussives, or bactericides. Other *Inula* spp. were described in Ayurvedic medicine and some ancient cultures for treating diseases such as inflammation, neoplasm, diabetes, and hypertension. This makes this genus a rich material for investigators [1]. *Iphiona* Cass. is another genus from the tribe *Inuleae* which holds some chemotaxonomical relation to *Inula*. *Inula salsoloides* Ostenf., also known as *Iphiona radiata,* has been reported to be used in treating fever and diuresis [1]. This evidence suggests that *Iphiona* Cass. might also have potential medicinal importance. Only two species of the *Iphiona* genus were recorded in the flora of Egypt, *Iphiona mucronata* (Forssk.) Asch. & Schweinf. and *Iphiona scabra* DC. The latter is a low glabrous annual shrub growing wildly in a stony desert, Wadis of inland desert and Sinai Peninsula of Egypt [2]. *I. mucronata* is widely distributed in the Eastern desert of Egypt but so far only two dehydrothymol derivatives were isolated from its aerial parts extract (ether, petroleum ether, and methanol, 1:1:1) [3] and the detailed phytochemical composition is unknown. Additionally, no previous pharmacological studies have been performed to assess its toxicity and its specific metabolites and biological activity have not yet been thoroughly investigated. Few reports showed that *Iphiona* Cass. had been considered a toxic plant for desert animals. *I. aucheri* was reported to intoxicate racing camels in the UAE, where two diterpene glycosides, atractylosides and carboxyatractylosides, have been implicated as the phytochemicals mainly contributing to its toxicity [4]. However, in another study, the methanolic and water extracts of *I. aucheri* exhibited low in vivo hepatotoxicity and caused no mortality as evaluated in mice, in contrast to the previous reports of being highly toxic to camels, rats, and sheep [5].

In the present study, the metabolite profile of *I. mucronata* aerial part was extensively studied for the first time using HRMS. Moreover, a toxicological evaluation of the plant alcohol extract has been carried out to better investigate the possible embryotoxicity in zebrafish and cytotoxicity in vitro. Cytotoxicity was evaluated by MTT assay to assess the cellular viability, which is proportional to the ability of cells to reduce the tetrazolium salt MTT to its insoluble formazan derivative [6]. Our results provide new insights into the unexplored potential of Saharan *I. mucronata* for the development and/or optimization of botanicals and pharmaceuticals.

## 2. Results and Discussion

### 2.1. Phytochemical Profiling

**Figure 1:**
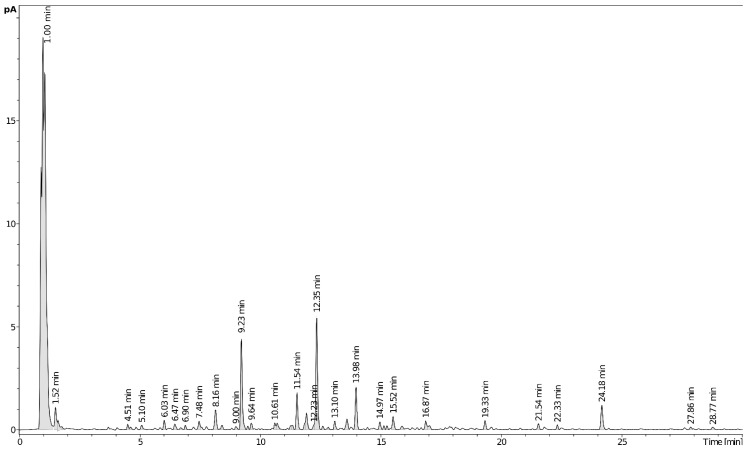
UHPLC-CAD profile of *Iphiona mucronata* ethanol extract.

**Figure 2:**
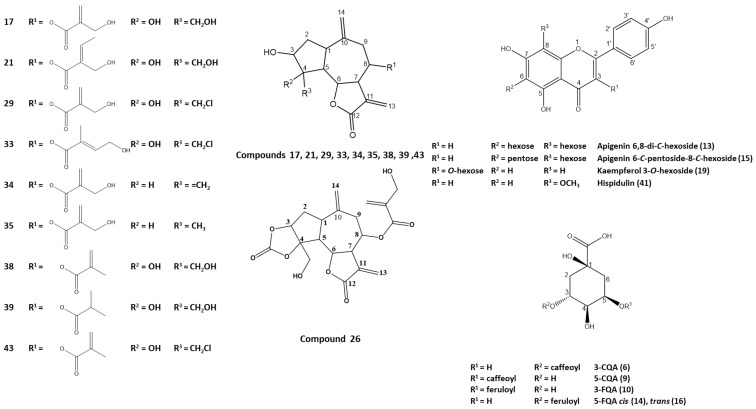
Structures of representative metabolites identified in the ethanolic extract of *Iphiona mucronata* aerial parts. Carbon numbering system for each compound is based on analogy rather than IUPAC rules. Metabolite numbers follow those listed in Table 1.

Metabolite profiling of plant extracts offers more insights into their complex phytochemical nature [7,8,9]. Chemical constituents of *I. mucronata* aerial parts were analyzed via UHPLC-PDA-CAD-ESI-Q-TOF-MS, which allowed for comprehensive chemical characterization of plant analytes (Table 1 and Supplementary Figures S1–S25). Extracts were analyzed in both positive and negative ion modes as genus *Iphiona* was reported to contain sesquiterpenes and flavonoids, which preferentially ionize under positive and negative ionization, respectively [10,11,12]. A total of 48 chromatographic peaks belonging to various metabolite classes were detected, including mostly sesquiterpenes, flavonoids, and phenolic acids (Table 1), of which only 3 flavonoids were previously identified in the genus *Iphiona*. A representative UHPLC–CAD chromatogram of *I. mucronata* extract is displayed in Figure 1. Structures of selected metabolites identified in the ethanol extract of *Iphiona mucronata* aerial parts are shown in Figure 2. To the best of our knowledge, this is the first comprehensive profile of *I. mucronata* secondary metabolites that provides chemical-based evidence for its biological effects. The following paragraphs describe the tentative identification of *I. mucronata*-specific metabolites.

#### 2.1.1. Flavonoids

Flavones and flavonols were shown to be the major subclass among flavonoids detected in *I. mucronata* extract. MS/MS fragmentation was employed to elucidate the structure of the metabolites, where there is a remarkable difference in the fragmentation pathways of the *O*-glycosyl and *C*-glycosyl flavonoids, which allows them to be easily differentiated. In the negative ion mode, for all of the *O*-glucosyl flavonoids, the most intense fragment results from the loss of the sugar unit, i.e., [M–H–162]^−^ (hexoses), [M–H–132]^−^ (pentoses), or [M–H–146]^−^ (deoxyhexose) [13]. However, the dominant fragmentation pathway of *C*-glucosyl flavonoids includes cross-ring cleavages [(O-C1 and C2-C3)] or [(O-C1 and C3-C4)] of the sugar units, namely [M–H–120/90]^−^ for *C*-hexoside, [M–H–90/60]^−^ for *C*-pentoside, and [M–H–104/74]^−^ for *C*-deoxyhexoside [14,15]. Additionally, fragment ions [Ag–H + 41/71]^−^ in mono-*C-* and [Ag–H + 83/113]^−^ for di-*C*-glycoside, which represent the aglycone (Ag), plus the remaining sugar residues linked to it, identified the type of aglycone [16]. 

Peak **13** [*m/z* 593.1512 (C_26_H_27_O_14_)^−^] shows typical fragmentation patterns of flavone-di-*C*-hexoside as evident from fragment ions at *m/z* 503 [M–H–90]^−^ and *m/z* 473 [M–H–120]^−^ (Supplementary Figure S1). Additionally, daughter ions at *m/z* 353 [Ag–H + 83]^−^ and 383 [Ag–H + 113]^−^ identified the aglycone as apigenin. Eventually, compound **13** was annotated as apigenin-6,8-*C*-di-hexoside [16].

Similarly, the MS/MS spectrum of peak **15** [*m/z* 563.1406 (C_26_H_27_O_14_)^−^] exhibits product ions at *m/z* 473 [M–H–90]^−^, *m/z* 443 [M–H–120]^−^, 383 [Ag–H + 113]^−^, and 353 [Ag–H + 83]^−^ (Supplementary Figure S2). Nevertheless, the appearance of a product ion at *m/z* 503 [M–H–60]^−^ characteristic for *C*-pentoside and the higher abundance of *C*-hexose fragment relative to *C*-pentose indicate hexose attachment at the C-6 position. All these findings confirmed the identification of compound **15** as apigenin 6-*C*-pentoside-8-*C*-hexoside [16].

The product ion spectrum of peak **19** [*m/z* 447.0933 (C_21_H_19_O_11_)^−^] showed a loss of the attached sugar unit and revealed a higher abundant ion at *m/z* 284 [Ag–2H]^−^, derived from a homolytic cleavage, relative to the corresponding ion at *m/z* 285 [Ag–H]^−^, derived from a heterolytic cleavage, respectively, (Supplementary Figure S3) and hence suggesting 3-*O*-glycosylation [17,18]. Consequently, compound **19** was annotated as kaempferol 3-*O*-hexoside.

Tetrahydroxyflavone *O*-diglycoside was detected in positive ion mode, peak **20** [*m/z* 595.1657 (C_27_H_31_O_15_)^+^], the high abundance of the fragment ion at *m/z* 449 due to loss of deoxyhexose [M + H–146]^+^ and subsequent loss of second sugar moiety, hexose [M + H–146–162]^+^, confirmed the attachment of the two sugar units to the flavone aglycone [19], and led to the identification of **20** as tetrahydroxyflavone-*O*-hexosyl-deoxyhexoside (Supplementary Figure S4). 

Peaks **22** [*m/z* 477.1038 (C_22_H_21_O_12_)^−^] and **30** [*m/z* 491.1195 (C_23_H_23_O_12_)^−^] are both methoxylated flavonoid derivatives. A product ion peak at *m/z* 462 and a base peak at *m/z* 315 (methoxy-tetrahydroxyflavone) formed after the elimination of a methyl group and a hexose moiety, respectively, identified compound **22** as methoxy-tetrahydroxyflavone-*O*-hexoside (Supplementary Figure S5). Similarly, the MS/MS spectrum of compound **30** showed product ions at *m/z* 476 and 461, due to successive losses of two methyl groups suggesting a methoxylated flavone derivative (dimethoxy-trihydroxyflavone), and another ion at *m/z* 329 [Ag–H]^−^ corresponding to the elimination of a hexose moiety confirmed the annotation of **30** as dimethoxy-trihydroxyflavone-*O*-hexoside (tricin-*O*-hexoside) [20].

Peak **24** [*m/z* 609.1814 (C_28_H_33_O_15_)]^+^ was annotated as trihydroxy-methoxy-flavone*-O*-deoxyhexosyl-hexoside (Table 1), showing a product ion at *m/z* 463 due to the loss of a sugar moiety or deoxyhexose, and a base peak at *m/z* 301 corresponding to loss of two sugar units [21].

Among other flavonoids detected were methoxylated aglycones in peaks **41** [*m/z* 299.0561 (C_16_H_11_O_6_)^−^] and **46** [*m/z* 329.1028 (C_18_H_17_O_6_)^+^]. Both compounds showed a similar fragmentation pattern, with product ions at *m/z* 284 and 314 derived from the loss of a methyl group, and were identified as methoxy-trihydroxyflavone and trimethoxy-hydroxyflavone, probably hispidulin and salvigenin, previously reported in genus *Iphiona* [22,23].

#### 2.1.2. Chlorogenic Acids

The fragmentation behavior of five detected chlorogenic acids has been investigated using LC-MS/MS analysis. Namely, two caffeoylquinic acid (CQA) isomers and three feruloylquinic acid (FQA) isomers could be discriminated against and annotated. These assignments were consistent and in agreement with the reported literature.

It is easy to distinguish 5-CQA (neochlorogenic) and 3-CQA (chlorogenic) acids by their base peaks at *m*/*z* 191 after the loss of a caffeoyl moiety. Moreover, it is possible to discriminate between the two isomers by a comparatively higher intense deprotonated caffeic acid ion at *m*/*z* 179 in neochlorogenic acid [24]. Accordingly, peaks **6** and **9** [*m/z* 353.0878 (C_16_H_17_O_9_)^−^] were identified as chlorogenic acid and neochlorogenic acid, respectively (Supplementary Figures S8 and S9).

Regarding FQAs in peaks **10**, **14**, and **16** [*m/z* 367.1035 (C_17_H_19_O_9_)^−^] (Supplementary Figures S10–S12), a base peak at *m/z* 193 corresponding to deprotonated ferulic acid is characteristic of 3-FQAs, while a base peak at *m/z* 191 due to loss of feruloyl moiety differentiates 5-FQA isomers [24]. This led to the identifications of compound **10** as 3-FQA and peaks **14** and **16** as 5-FQAs. It was previously reported that *cis*-5-acyl chlorogenic acids, being comparatively more hydrophobic, elute appreciably later from reversed-phase column packings than their *trans* counterparts [25]. Thus, compounds **14** and **16** were identified as *trans*-5- FQA and *cis*-5- FQA, respectively.

#### 2.1.3. Sesquiterpene Lactones (Guaianolides)

A total of 17 sesquiterpene lactones, with guaianolides representing the main class, were detected in the *I. mucronata* extract and were preferentially ionized under positive ion mode. The structures of identified guaianolides are displayed in Figure 2, with 3-hydroxy-guai-10(14) and 11(13)-dien-12,6-olide (GUAI) representing the main backbone.

A discussion is given of the fragmentation processes of 14 guaianolides with voluminous substituents at C-8, thus causing instability of the molecular ions. The compositions of the fragment ions have been determined, and it has been shown that the breakdown of the lactone skeleton takes place only after the elimination of these voluminous substituents at C8, i.e., (M –R_2_OH)^+^ [26]. 

In the present paper, we consider compounds of the GUAI series (Figure 2) with a voluminous substituent at C-8, namely, angelic [116 amu], 3-hydroxy-isobutyric [104 amu], 2-hydroxymethyl-acrylic [102 amu], isobutyric [88 amu], and methacrylic [86 amu] acids, and with hydroxyl, chloromethyl, hydroxymethyl, methyl, or methylene groups at C-4 [27,28,29]. The subsequent detachment of small fragments (H_2_O, ^•^CH_2_Cl, ^•^Cl) from the lactone nucleus after the elimination of the voluminous ROH was also observed in the spectra (Supplementary Figures S1–25).

Compounds **17** [*m/z* 381.1544 (C_19_H_25_O_8_)^+^], **21** [*m/z* 395.1700 (C_20_H_27_O_8_)^+^], **38** [*m/z* 365.1595 (C_19_H_25_O_7_)^+^], and **39** [*m/z* 367.1751 (C_19_H_27_O_7_)^+^] showed a distinct fragment ion at *m/z* 279 corresponding to a guaianolide substituted with a hydroxyl and hydroxymethyl groups at C-4. In detail, such an ion was formed after losses of 2-hydroxymethyl-acrylic acid [M + H–102]^+^ in peak **17**, angelic acid [M + H–116]^+^ in **21**, methacrylic acid [M + H–86]^+^ in **38,** and isobutyric acid [M + H–88]^+^ in guaianolide **39** (Supplementary Figures S13–S16). Accordingly, compounds **17**, **21**, **38,** and **39** were identified as 15-hydroxy-janerin, 8-angeloyloxy-4-hydroxy-4-hydroxymethyl-GUAI, 8-methacryoyloxy-4-hydroxy-4-hydroxymethyl-GUAI, and 8-isobutyroyloxy-4-hydroxy-4-hydroxymethyl-GUAI, respectively [30].

Similar fragmentation was also observed in compounds **23** [*m/z* 465.1755 (C_23_H_29_O_10_)^+^], **25/27** [*m/z* 423.1650 (pattern C_21_H_27_O_9_)^+^], and **31** [*m/z* 437.1806 (C_22_H_29_O_9_)^+^] all showing fragment ion at *m/z* 279, yet with extra loss of acetyl group(s) [42 amu] after the elimination of R_2_OH, i.e., [M + H–102–2 × 42]^+^, [M + H–102–42]^+^, and [M + H–116–42]^+^ in compounds **23**, **25/27**, and **31**, respectively (Supplementary Figures S17–S19). Guaianolides **23**, **25/27**, and **31** were thus annotated as 15-hydroxy-janerin diacetate, 15-hydroxy-janerin acetate, and 8-angeloyloxy-4-hydroxy-4-hydroxymethyl-GUAI acetate, respectively.

Compounds **34** [*m/z* 347.1489 (C_19_H_23_O_6_)^+^] and **35** [*m/z* 366.1911 (C_19_H_28_NO_6_)^+^ as ammonium adduct] revealed a fragment ion at *m/z* 245 and 247, respectively, characteristic of a guaianolide substituted with an exocyclic methylene and a methyl group at C-4, respectively. Such ions were formed after splitting out of 2-hydroxymethyl-acrylic acid [M + H–102]^+^ in both compounds (Supplementary Figures S20 and S21) and led to the identification of compounds **34** and **35** as 8-(2-hydroxymethyl)acryloyloxy-4-methylene-GUAI (cynaropicrin) and 8-(2-hydroxymethyl)acryloyloxy-4-methyl-GUAI, respectively [30,31]. Notably, other sesquiterpene acetates as represented by eudesmol xylopyranosides acetates were previously reported from *I. mucronata* polar fraction and thus the acetate derivatives could be of chemotaxomomic importance for the Genus *Iphiona* [32].

Chlorinated guaianolides were also identified in *I. mucronata* extract, namely chlorojanerin (**29**) [*m/z* 399.1205 (C_19_H_24_ClO_7_)^+^], cebellin D (**33**) [*m/z* 413.1362 (C_20_H_26_ClO_7_)^+^], and linichlorin A (**43**) [*m/z* 383.1256 (C_19_H_24_ClO_6_)^+^] (Supplementary Figures S22–S24). All chlorinated guaianolides showed a fragment ion at *m/z* 297, corresponding to a guaianolide substituted with hydroxyl and chloromethyl groups at C-4, after the loss of 2-hydroxymethyl-acrylic [M + H–102]^+^, hydroxyangelic [M + H–116]^+^, and methacrylic [M + H–86]^+^ acids, in peaks **29**, **33**, and **43**, respectively [30].

Among other guaianolides identified was hololeucin or peak **26** [*m/z* 407.1337 (C_20_H_23_O_9_)^+^] showing a fragment ion at *m/z* 305, revealing a hydroxyl group and a cyclic carbonate at C-4, after the loss of 2-hydroxymethyl-acrylic acid [M + H–102]^+^ (Supplementary Figure S25). Another ion at *m/z* 243, possibly due to loss of CO_2_ and H_2_O [M + H–102–44–18]^+^ after cleavage of cyclic carbonate, confirms the structure [33].

### 2.2. Zebrafish Toxicity

Zebrafish embryo toxicity model is a new approach method that might overcome cell- and protein-based assays and become a substitute for mammalian testing. The development of zebrafish embryos might be perturbed by tested chemicals and could be manifested in morphological malformations, behavioral abnormalities, or the death of the embryos. The model gives many advantages, fast development and transparency of embryos allow to monitor any sign of toxicity already on the cell level using microscope techniques [39,40]. 

It was shown that *I. mucronata* extract has no toxic effects at low concentrations tested (5–40 and 50, 75 µg/mL). The first sign of toxicity was noticed after 72 h incubation at the concentration of 100 µg/mL when all 10 larvae exhibited a slow touch response after a poke at the end of the tail, which might be a symptom of a disturbed peripheral nervous system. Certain sesquiterpenes characterized by Asteraceae plants were previously evaluated for zebrafish embryotoxicity. For instance, an artemisinin metabolite; dihydroartemisinin (DHA) (1–10 mg/L) caused abnormality in embryonic phenotypes, while 10 mg/L of DHA also affected the developmental zebrafish embryo by increasing angiogenesis [41]. On the other hand, the effect of flavonoids on zebrafish embryos is widely varied. In a comparative study by Bugel et al., [42], 15 out of 24 investigated flavonoids evoked negative effects on the tested developmental or behavioral endpoints of zebrafish embryos at concentrations of 1–50 µM. 

### 2.3. Cytotoxicity and Antineoplastic Selectivity

**Figure 3:**
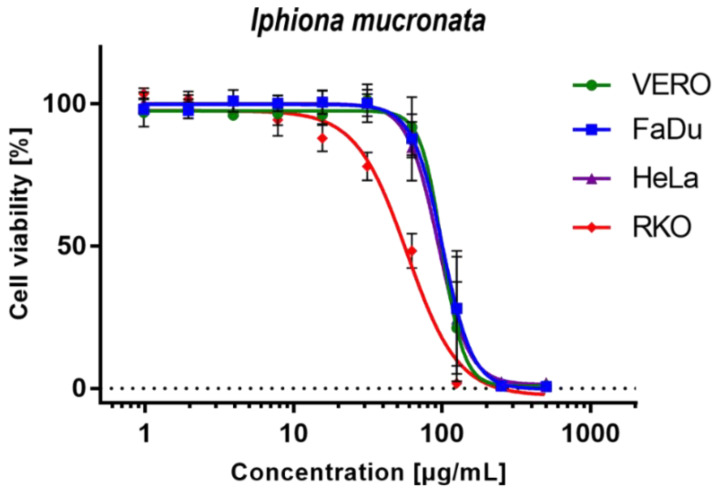
Dose–response influence of *Iphiona mucronata* ethanolic extract on selected cell lines.

The results of cytotoxicity testing presented in Table 2 show that *I. mucronata* ethanolic extract exerts similar toxicity towards normal VERO cells and two of the cancer cell lines—hypopharyngeal squamous cell carcinoma and cervical adenocarcinoma, with CC_50_ values between 91.6 and 101.7 µg/mL. Moreover, dose–response curves shown in Figure 3 exhibit similar patterns for VERO, FaDu, and HeLa cells. However, statistically significant (*p* < 0.05) antineoplastic selectivity (SI = 1.84) was observed in human colon carcinoma (RKO) cells with CC_50_ of 54.5 µg/mL. To the best of our knowledge, this is the first report on the in vitro cytotoxicity of *I. mucronata*. Interestingly, the review of available literature showed the absence of any information concerning cytotoxicity studies of other plants belonging to the genus *Iphiona*. The observed cytotoxic effect could be owed to the enriched profile with guaianolides, particularly chlorinated ones. Previous studies report on the potential of these sesquiterpenes to induce cytotoxic activity. Sary et al. [43] isolated two chlorinated guaianolides; cenegyptin A and cenegyptin B, from the aerial parts of *Centaurea aegyptiaca*, where the first compound showed a potent cytotoxic effect against HEPG2 and HEP2 cell lines (IC_50_ = 7.2 ± 0.04 and 7.5 ± 0.02 µM). Notably, other chlorinated guaianolides; including chlorojanerin (also identified in the current study) and 19-deoxychlorojanerin, showed high cytotoxic activity against MDA-MB-231 cell lines (IC_50_; 2.21 and 2.88 µM, respectively) [30].

## 3. Materials and Methods

### 3.1. Plant Material

The aerial parts of *Iphiona mucronata* (Forssk.) Asch. & Schweinf., as a composite sample, were collected during the flowering stage (March 2020) in plastic bags from Wadi Arabah, Northeastern Desert (29°1′23.20” N 32°10′9.42” E), Egypt. The collected plant was identified according to Boulos (2002) and Tackholm (1974) [2]. Plant material was cleaned of any impurities and air-dried at room temperature (25 ± 3 °C) in shade for 7 days. A voucher specimen (Mans. 0010913004) was prepared and deposited in the Herbarium of Botany Department, Faculty of Science, Mansoura University, Mansoura, Egypt.

### 3.2. Preparation of Plant Extract

A total of 100 g of the powdered plant material was extracted by maceration for 10 days using 70% ethanol till complete exhaustion. The filtered extract was then evaporated to dryness at 50 °C using a rotary evaporator. The dried extract was kept at −80 °C for further chemical and biological analysis.

### 3.3. LC-MS and Qualitative Analysis

The extract was analyzed using a high-resolution LC-MS Thermo Scientific Ultimate 3000RS chromatographic system. The separation was carried out on a Waters Acquity HSS T3 column (150 × 2.1 mm i.d.; 1.8 µm, Milford, CT, USA) at 45 °C using a linear gradient from 5% to 70% phase B (acetonitrile with 0.1% formic acid) in phase A (0.1% formic acid in Milli-Q water) for 30 min, with a flow rate of 0.4 mL/min. 

The photodiode array detector recorded absorbances in the 190–600 nm wavelength range with 5 nm bandwidth and 10 Hz acquisition frequency. A flow splitter was used to divert the column effluent in a proportion of 1:3 between qTOF MS (Bruker Impact II HD, Bruker, Billerica, MA, USA) and charged aerosol detector (CAD, Thermo Corona Veo RS) linked in parallel. The acquisition frequency for CAD was 10 Hz.

The MS analyses were operated in both positive and negative ion modes, using electrospray ionization. Linear spectra were obtained in the *m/z* 80 to *m/z* 1800 mass range, with 5 Hz acquisition frequency and the following parameters of the mass spectrometer: negative ion capillary voltage 3.0 kV; positive ion capillary voltage 4.0 kV; dry gas flow 6 L/min; dry gas temperature 200 °C; collision cell transfer time 90 μs; nebulizer pressure 0.7 bar. The obtained data were calibrated internally with sodium formate introduced into the ion source via a 20 µL loop at the start of each separation. The chromatographic data were acquired and processed using Bruker DataAnalysis 4.4 software, and metabolite structure elucidation and identification were achieved mostly using SIRIUS 4.8.2 software integrating CSI: FingerID for searching in molecular structure databases [44,45].

### 3.4. Zebrafish Embryo Toxicity (ZET) Assay

Zebrafish (*Danio rerio*) stocks of the AB strain were maintained at 28.5 °C on a 14/10 h light/dark cycle under standard aquaculture conditions, and fertilized eggs were collected via natural spawning. Embryos were reared under 14/10 h light/dark conditions in embryo medium: 1.5 mM HEPES, pH 7.1–7.3, 17.4 mM NaCl, 0.21 mM KCl, 0.12 mM MgSO_4_, and 0.18 mM Ca(NO_3_)_2_ at 28.5 °C. The 4-hpf embryos were placed in 48-well plates, 5 embryos per well, and then incubated in 4 different concentrations of tested extract—10 embryos per concentration (n = 10). After 24, 48, and 72 h, the embryos were checked under the microscope for any signs of cytotoxicity such as coagulation of the embryo, a lack of somite formation, non-detachment of the tail, and/or a lack of heartbeat. Each day extract solution was changed. Two ranges of concentrations were tested, first 20–50 μg/mL and then 40–100 μg/mL. 

### 3.5. Cell Lines Maintenance, Cytotoxicity Testing, and Antineoplastic Selectivity

The media for cell culturing (MEM and DMEM), antibiotics (penicillin-streptomycin, 100-fold working concentration), phosphate-buffered saline (PBS), and trypsin were purchased from Corning (Tewksbury, MA, USA), and fetal bovine serum (FBS) from Capricorn Scientific (Ebsdorfergrund, Germany). Sodium dodecyl-sulphate (SDS) was acquired from PanReac Applichem (Darmstadt, Germany), dimethyl sulfoxide (DMSO, p.a.), and dimethylformamide (DMF) from Avantor Performance Materials (Gliwice, Poland), while 3-(4,5-dimethylthiazol-2-yl)-2,5-diphenyltetrazolium bromide (MTT) from Sigma-Aldrich (St. Louis, MI, USA). The VERO cells were cultured using DMEM, whereas FaDu, HeLa, and RKO using MEM. Cell lines were incubated at 37 °C in a 5% CO_2_ (Panasonic Healthcare Co., Ltd., Tokyo, Japan). Stock solutions (50 mg/mL) of *Iphiona mucronata* ethanolic extract were prepared by dissolving the extract in cell-culture grade DMSO (PanReac Applichem).

The cytotoxicity testing was carried out on VERO (ECACC, 84113001, kidney of a normal adult African Green monkey), FaDu (ATCC, HTB-43, human hypopharyngeal squamous cell carcinoma), HeLa (ECACC 93021013, human cervical adenocarcinoma), and RKO (ATCC CRL-2577, human colon carcinoma) cell lines using MTT assay as previously described [46]. In short, selected cell lines seeded in 96-well plates were incubated with serial dilution (500–1 µg/mL) of *Iphiona mucronata* extract stock solution in cell media for 72 h. Cells supplemented with complete media were used as a non-treated control. Subsequently, extract containing cell media was discarded, plates were washed with PBS, cell media with MTT was added, and plates were further incubated for 4 h. Finally, to dissolve the violet formazan product, the SDS/DMF/DMSO-based solvent was added, and the plates were incubated overnight. The absorbance (540 and 620 nm) was measured using Synergy H1 Multi-Mode Microplate Reader (BioTek Instruments, Inc. Winooski, VT, USA). The GraphPad Prism (version 7.04) software was used for data analysis, and the CC_50_ values (concentrations decreasing the cellular viability by 50%) were calculated from dose–response curves. The CC_50_ values were expressed as mean ± SD (*n* ≥ 3). The antineoplastic activity was evaluated based on selectivity indexes (SI; SI = CC_50_VERO/CC_50_Cancer), where SI > 1 indicates selectivity. 

### 3.6. Statistical Analysis

GraphPad Prism software was used for statistical analysis. The CC_50_ values calculated for cancer cell lines were compared with CC_50_ recorded for VERO cells using one-way ANOVA with Dunnett’s post hoc test of significance, where *p* < 0.05 was recognized as statistically significant. 

## 4. Conclusions

The detailed metabolite profiling was investigated for the underexplored *Iphiona mucronata*. A total of 48 constituents were putatively identified representing sesquiterpenes, flavonoids, and phenolic acids. To the best of our knowledge, this is the first comprehensive profile of *I. mucronata* secondary metabolites that provides chemical-based evidence for its biological effects. Possible neurological effects are implicated in zebrafish embryotoxicity testing. The in vitro cytotoxicity of *I. mucronata* has been reported for the first time, and results indicate selective antineoplastic activity toward human colon carcinoma cells. However, further studies are necessary to elucidate the compounds responsible for this activity. The enriched sesquiterpene and flavonoid profile warrants further phytochemical investigations to valorize its pharmaceutical uses.

## Figures and Tables

**Table 1:** Compounds identified in the ethanolic extract of *Iphiona mucronata* aerial parts using UHPLC-qTOF-MS/MS.

No.	Compound Name	RT (min)	Neutral Formula	Error (ppm)	mσ	Measured *m/z* (Formula)	Major Fragments *m/z* (Formula)	Reference
1.	Mixture of polar constituents	0.8–1.4	-	-	-	-	-	-
2.	*N*-Fructosyl pyroglutamate	1.52	C_11_H_17_NO_8_	0.0	15.3	290.0881 (C_11_H_16_NO_8_^−^)	**200.0564** (C_8_H_10_NO_5_^−^), 128.0353 (C_5_H_6_NO_3_^−^)	-
3.	Adenosine	1.62	C_10_H_13_N_5_O_4_	−0.4	23.1	268.1045 (C_10_H_14_N_5_O_4_^+^)	**268.1041** (C_10_H_14_N_5_O_4_^+^), 136.0617 (C_4_H_10_NO_4_^+^)	-
4.	*N*-Fructosyl (iso)leucine	1.78	C_12_H_23_NO_7_	−1.6	14.0	294.1552 (C_12_H_24_NO_7_^+^)	**276.1442** (C_12_H_22_NO_6_^+^), 258.1336 (C_12_H_20_NO_5_^+^), 248.1492 (C_11_H_22_NO_5_^+^), 230.1387 (C_11_H_20_NO_4_^+^)	-
5.	Erigeside C [1-*O*-(4-hydroxy-3,5-dimethoxybenzoyl-hexose]	4.51	C_15_H_20_O_10_	−1.9	3.7	359.0984 (C_15_H_19_O_10_^−^)	**197.0455** (C_9_H_9_O_5_^−^), 182.0221 (C_8_H_6_O_5_^−^), 153.0557 (C_8_H_9_O_3_^−^), 138.0322 (C_7_H_6_O_3_^−^)	[34]
6.	3-CQA	4.63	C_16_H_18_O_9_	−2.1	2.3	353.0878 (C_16_H_17_O_9_^−^)	**191.0561** (C_7_H_11_O_6_^−^), 179.0350 (C_9_H_7_O_4_^−^), 173.0455 (C_7_H_9_O_5_^−^), 135.0452 (C_8_H_7_O_2_^−^)	[24]
7.	Danielone [3′,5′-Dimethoxy-4′-hydroxy-(2-hydroxy)acetophenone]	4.68	C_10_H_12_O_5_	−2.4	11.3	211.0612 (C_10_H_11_O_5_^−^)	196.0377 (C_9_H_8_O_5_^−^), **181.0506** (C_9_H_9_O_4_^−^), 166.0272 (C_8_H_6_O_4_^−^), 163.0401 (C_9_H_7_O_3_^−^)	[35]
8.	Picein (4-acetylphenyl hexoside)	5.10	C_14_H_18_O_7_	−2.3	65.8	343.1035 * (C_15_H_19_O_9_^−^)	**135.0452** (C_8_H_7_O_2_^−^)	[36]
9.	5-CQA	6.03	C_16_H_18_O_9_	−2.7	3.8	353.0878 (C_16_H_17_O_9_^−^)	191.0561 (C_7_H_11_O_6_^−^), **179.0350** (C_9_H_7_O_4_^−^), 161.0244 (C_9_H_5_O_3_^−^)	[24]
10.	3-FQA	6.47	C_17_H_20_O_9_	−2.3	3.5	367.1035 (C_17_H_19_O_9_^−^)	**193.0506** (C_10_H_9_O_4_^−^), 191.0561 (C_7_H_11_O_6_^−^), 134.0373 (C_8_H_6_O_2_^−^)	[24]
11.	Unidentified	6.71	C_14_H_20_O_8_	−2.3	14.4	315.1093 (C_14_H_19_O_8_^−^)	-	-
12.	Unidentified	6.90	C_24_H_33_NO_10_	−2.3	5.6	494.2032 (C_24_H_32_NO_10_^−^)	**114.0565** (C_5_H_8_NO_2_^−^)	-
13.	Apigenin 6,8-di-*C*-hexoside	7.48	C_27_H_30_O_15_	−3.0	9.2	593.1512 (C_27_H_29_O_15_^−^)	503.1195 (C_24_H_23_O_12_^−^), 473.1089 (C_23_H_21_O_11_^−^), 383.0772 (C_20_H_15_O_8_^−^), **353.0667** (C_19_H_13_O_7_^−^)	[16]
14.	*trans*-5-FQA	8.16	C_17_H_20_O_9_	−1.9	2.9	367.1035 (C_17_H_19_O_9_^−^)	193.0506 (C_10_H_9_O_4_^−^), **191.0561** (C_7_H_11_O_6_^−^), 173.0455 (C_7_H_9_O_5_^−^), 134.0373 (C_8_H_6_O_2_^−^)	[24,25]
15.	Apigenin 6-*C*-pentoside-8-*C*-hexoside	8.42	C_26_H_28_O_14_	−3.3	5.3	563.1406 (C_26_H_27_O_14_^−^)	545.1301 (C_26_H_25_O_13_^−^), 503.1195 (C_24_H_23_O_12_^−^), 473.1089 (C_23_H_21_O_11_^−^), 443.0984 (C_22_H_19_O_10_^−^), 383.0772 (C_20_H_15_O_8_^−^), **353.0667** (C_19_H_13_O_7_^−^)	[16]
16.	*cis*-5-FQA	9.00	C_17_H_20_O_9_	−2.3	4.4	367.1035 (C_17_H_19_O_9_^−^)	**191.0561** (C_7_H_11_O_6_^−^), 173.0455 (C_7_H_9_O_5_^−^)	[24,25]
17.	15-Hydroxy-janerin	9.23	C_19_H_24_O_8_	−2.4	2.9	381.1544 (C_19_H_25_O_8_^+^)	363.1438 (C_19_H_23_O_7_^+^), 279.1227 (C_15_H_19_O_5_^+^), **261.1121** (C_15_H_17_O_4_^+^), 243.1016 (C_15_H_15_O_3_^+^), 225.0910 (C_15_H_13_O_2_^+^)	[30]
18.	Unidentified	9.49	C_11_H_16_O_3_	−1.4	14.1	197.1172 (C_11_H_17_O_3_^+^)	**179.1067** (C_11_H_15_O_2_^+^), 161.0961 (C_11_H_13_O^+^), 135.1168 (C_10_H_15_^+^)	-
19.	Kaempferol 3-*O*-hexoside	9.64	C_21_H_20_O_11_	−2.5	24.9	447.0933 (C_21_H_19_O_11_^−^)	**284.0326** (C_15_H_8_O_6_^−^)	[18]
20.	Tetrahydroxyflavone-*O*-hexosyl-deoxyhexoside	9.64	C_27_H_30_O_15_	−2.1 −2.1	11.46.8	593.1524 (C_27_H_29_O_15_^−^) 595.1657 (C_27_H_31_O_15_^+^)	**285.0411** (C_15_H_9_O_6_^−^) 449.1078 (C_21_H_21_O_11_^+^), **287.0550** (C_15_H_11_O_6_^+^)	[19]
21.	8-Angeloyloxy-4-hydroxy-4-hydroxymethyl-GUAI	10.61	C_20_H_26_O_8_	−1.0	5.2	395.1700 (C_20_H_27_O_8_^+^)	279.1227 (C_15_H_19_O_5_^+^), 261.1121 (C_15_H_17_O_4_^+^), **243.1016** (C_15_H_15_O_3_^+^), 225.0910 (C_15_H_13_O_2_^+^), 215.1067 (C_14_H_15_O_2_^+^), 197.0961 (C_14_H_13_O^+^)	-
22.	Methoxy-tetrahydroxyflavone-O-hexoside	10.71	C_22_H_22_O_12_	−1.7	8.5	477.1038 (C_22_H_21_O_12_^−^)	462.0804 (C_21_H_18_O_12_^−^), 357.0616 (C_18_H_13_O_8_^−^), **315.0510** (C_16_H_11_O_7_^−^), 299.0197 (C_15_H_7_O_7_^−^), 272.0326 (C_14_H_8_O_6_^−^)	-
23.	15-Hydroxy-janerin diacetate	11.27	C_23_H_28_O_10_	−0.9	4.8	465.1755 (C_23_H_29_O_10_^+^)	447.1650 (C_23_H_27_O_9_^+^), 345.1333 (C_19_H_21_O_6_^+^), 279.1227 (C_15_H_19_O_5_^+^), **261.1121** (C_15_H_17_O_4_^+^), 243.1016 (C_15_H_15_O_3_^+^), 225.0910 (C_15_H_13_O_2_^+^)	-
24.	Trihydroxy-methoxy-flavone *O*-deoxyhexosyl-hexoside	11.34	C_28_H_32_O_15_	−0.9 −2.6	38.129.1	607.1674 (C_28_H_31_O_15_^−^) 609.1814 (C_28_H_33_O_15_^+^)	**299.0562** (C_16_H_11_O_6_^−^), 284.0323 (C_15_H_8_O_6_^−^) 463.1235 (C_22_H_23_O_11_^+^), **301.0708** (C_16_H_13_O_6_^+^)	[21]
25.	15-Hydroxy-janerin acetate	11.54	C_21_H_26_O_9_	−0.7	6.2	423.1650 (C_21_H_27_O_9_^+^)	405.1544 (C_21_H_25_O_8_^+^), 303.1227 (C_17_H_19_O_5_^+^), 279.1227 (C_15_H_19_O_5_^+^), 261.1121 (C_15_H_17_O_4_^+^), **243.1016** (C_15_H_15_O_3_^+^), 225.0910 (C_15_H_13_O_2_^+^)	-
26.	Hololeucin	11.86	C_20_H_22_O_9_	−0.8	10.7	407.1337 (C_20_H_23_O_9_^+^)	305.1020 (C_16_H_17_O_6_^+^), 287.0914 (C_16_H_15_O_5_^+^), 243.1016 (C_15_H_15_O_3_^+^), 225.0910 (C_15_H_13_O_2_^+^), **215.1067** (C_14_H_15_O_2_^+^), 197.0961 (C_14_H_13_O^+^)	[33]
27.	15-Hydroxy-janerin acetate	11.93	C_21_H_26_O_9_	−0.9	8.4	423.1650 (C_21_H_27_O_9_^+^)	405.1544 (C_21_H_25_O_8_^+^), 345.1333 (C_19_H_21_O_6_^+^), 305.1020 (C_16_H_17_O_6_^+^), 261.1121 (C_15_H_17_O_4_^+^), **243.1016** (C_15_H_15_O_3_^+^), 225.0910 (C_15_H_13_O_2_^+^)	-
28.	Secoisolariciresinol	12.27	C_20_H_26_O_6_	−0.4	61.5	361.1509 (C_20_H_25_O_6_^−^)	**346.1274** (C_19_H_22_O_6_^−^), 165.0477 (C_9_H_9_O_3_^−^)	[37]
29.	Chlorojanerin	12.35	C_19_H_23_ClO_7_	0.3	5.3	399.1205 (C_19_H_24_ClO_7_^+^)	**279.0782** (C_15_H_16_ClO_3_^+^), 261.0677 (C_15_H_14_ClO_2_^+^), 233.0728 (C_14_H_14_ClO^+^), 201.0677 (C_10_H_14_ClO_2_^+^)	[30]
30.	Dimethoxy-trihydroxyflavone-*O*-hexoside (tricin-*O*-hexoside)	12.60	C_23_H_24_O_12_	−0.9	9.7	491.1195 (C_23_H_23_O_12_^−^)	476.0960 (C_22_H_20_O_12_^−^), 461.0725 (C_21_H_17_O_12_^−^), 329.0667 (C_17_H_13_O_7_^−^), **313.0354** (C_16_H_9_O_7_^−^), 299.0197 (C_15_H_7_O_7_^−^), 285.0405 (C_15_H_9_O_6_^−^),	[20]
31.	8-Angeloyloxy-4-hydroxy-4-hydroxymethyl-GUAI acetate	12.83	C_22_H_28_O_9_	−0.9	23.4	437.1806 (C_22_H_29_O_9_^+^)	419.1700 (C_22_H_27_O_8_^+^), 303.1227 (C_17_H_19_O_5_^+^), 279.1227 (C_15_H_19_O_5_^+^), 261.1121 (C_15_H_17_O_4_^+^), **243.1016** (C_15_H_15_O_3_^+^), 225.0910 (C_15_H_13_O_2_^+^)	-
32.	Unidentified sesquiterpenoid lactone	13.10	C_38_H_48_O_16_	−0.8	8.7	761.3015 (C_38_H_49_O_16_^+^)	707.2698 (C_38_H_43_O_13_^+^), 429.1544 (C_23_H_25_O_8_^+^), **363.1438** (C_19_H_23_O_7_^+^), 345.1333 (C_19_H_21_O_6_^+^), 279.1227 (C_15_H_19_O_5_^+^), 243.1016 (C_15_H_15_O_3_^+^),	-
33.	Cebellin D	13.61	C_20_H_25_ClO_7_	−1.4	11.6	413.1362 (C_20_H_26_ClO_7_^+^)	297.0888 (C_15_H_18_ClO_4_^+^), **279.0782** (C_15_H_16_ClO_3_^+^), 261.0677 (C_15_H_14_ClO_2_^+^), 233.0728 (C_14_H_14_ClO^+^), 183.0571 (C_10_H_12_ClO^+^)	-
34.	Cynaropicrin	13.98	C_19_H_22_O_6_	−1.0	1.4	347.1489 (C_19_H_23_O_6_^+^)	245.1172 (C_15_H_17_O_3_^+^), **227.1067** (C_15_H_15_O_2_^+^), 217.1223 (C_14_H_17_O_2_^+^), 199.1117 (C_14_H_15_O^+^), 181.1012 (C_14_H_13_^+^)	[30]
35.	8-(2-hydroxymethyl)acryloyloxy-4-methyl-GUAI	14.97	C_19_H_24_O_6_	−1.8	21.1	366.1911 ** (C_19_H_28_NO_6_^+^)	247.1329 (C_15_H_19_O_3_^+^), **229.1223** (C_15_H_17_O_2_^+^), 211.1117 (C_15_H_15_O^+^), 201.1274 (C_14_H_17_O^+^), 183.1168 (C_14_H_15_^+^)	[31]
36.	Unidentified sesquiterpenoid lactone	15.14	C_38_H_47_ClO_15_	−1.3	40.3	779.2676 (C_38_H_48_ClO_15_^+^)	587.2276 (C_34_H_3_O_9_^+^), 429.1544 (C_23_H_25_O_8_^+^), 399.1205 (C_19_H_24_ClO_7_^+^), **381.1121** (C_25_H_17_O_4_^+^), 363.1016 (C_25_H_15_O_3_^+^), 345.1333 (C_19_H_21_O_6_^+^), 279.0782 (C_15_H_16_ClO_3_^+^), 261.1121 (C_15_H_17_O_4_^+^), 233.0728 (C_14_H_14_ClO^+^), 225.0910 (C_15_H_13_O_2_^+^)	-
37.	Unidentified sesquiterpenoid lactone	15.26	C_38_H_46_O_15_	−1.3	12.2	743.2909 (C_38_H_47_O_15_^+^)	345.1333 (C_19_H_21_O_6_^+^), 279.1227 (C_15_H_19_O_5_^+^), **261.1121** (C_15_H_17_O_4_^+^), 243.1016 (C_15_H_15_O_3_^+^), 225.0910 (C_15_H_13_O_2_^+^), 197.0961 (C_14_H_13_O^+^)	-
38.	8-Methacryoyloxy-4-hydroxy-4-hydroxymethyl-GUAI	15.52	C_19_H_24_O_7_	−2.1	3.2	365.1595 (C_19_H_25_O_7_^+^)	279.1227 (C_15_H_19_O_5_^+^), 261.1121 (C_15_H_17_O_4_^+^), **243.1016** (C_15_H_15_O_3_^+^), 225.0910 (C_15_H_13_O_2_^+^), 215.1067 (C_14_H_15_O_2_^+^), 197.0961 (C_14_H_13_O^+^)	-
39.	8-Isobutyroyloxy-4-hydroxy-4-hydroxymethyl-GUAI	15.89	C_19_H_26_O_7_	−2.7	20.6	367.1751 (C_19_H_27_O_7_^+^)	279.1227 (C_15_H_19_O_5_^+^), **261.1121** (C_15_H_17_O_4_^+^), 243.1016 (C_15_H_15_O_3_^+^), 231.1016 (C_14_H_15_O_3_^+^), 215.1067 (C_14_H_15_O_2_^+^)	-
40.	*N*,*N*,*N*,*N*-Tetra-*p*-coumaroyl-spermine	16.87	C_46_H_50_N_4_O_8_	2.0	5.4	787.3524 (C_46_H_51_N_4_O_8_^+^)	**641.3199** (C_37_H_45_N_4_O_6_^+^), 623.3098 (C_37_H_43_N_4_O_5_^+^), 478.2612 (C_28_H_38_N_4_O_3_^+^)	[38]
41.	Methoxy-trihydroxyflavone (hispidulin)	16.99	C_16_H_12_O_62_	3.1	12.9	299.0561 (C_16_H_11_O_6_^−^)	**284.0326** (C_15_H_8_O_6_^−^), 227.0350 (C_13_H_17_O_4_^−^)	-
42.	Unidentified sesquiterpenoid lactone	17.04	C_38_H_46_O_15_	−1.3	8.2	743.2909 (C_38_H_47_O_15_^+^)	689.2593 (C_38_H_41_O_12_^+^), 447.1650 (C_23_H_27_O_9_^+^), 429.1544 (C_23_H_25_O_8_^+^), 411.1438 (C_23_H_23_O_7_^+^), 279.1227 (C_15_H_19_O_5_^+^), 261.1121 (C_15_H_17_O_4_^+^), **243.1016** (C_15_H_15_O_3_^+^), 225.0910 (C_15_H_13_O_2_^+^), 197.0961 (C_14_H_13_O^+^), 187.0601 (C_8_H_11_O_5_^+^), 169.0495 (C_8_H_9_O_4_^+^)	-
43.	Linichlorin A	19.33	C_19_H_23_ClO_6_	−2.6	27.5	383.1256 (C_19_H_24_ClO_6_^+^)	297.0888 (C_15_H_18_ClO_4_^+^), **279.0782** (C_15_H_16_ClO_3_^+^), 261.0677 (C_15_H_14_ClO_2_^+^), 233.0728 (C_14_H_14_ClO^+^), 183.0571 (C_10_H_12_ClO^+^)	[30]
44.	Unidentified	21.54	C_37_H_38_O_15_	2.8	16.2	721.2138 (C_37_H_37_O_15_^−^)	660.1848 (C_35_H_32_O_13_^−^), 645.1614 (C_34_H_29_O_13_^−^), 573.1766 (C_32_H_29_O_10_^−^), **313.0718** (C_17_H_13_O_6_^−^), 298.0483 (C_16_H_10_O_6_^−^), 283.0248 (C_22_H_30_O_9_^−^)	-
45.	Unidentified	22.33	C_29_H_40_O_18_	5.4	30.0	677.2287 (C_29_H_41_O_18_^+^)	659.2182 (C_29_H_39_O_17_^+^), 575.1970 (C_25_H_35_O_15_^+^), 557.1865 (C_25_H_33_O_14_^+^), **315.0922** (C_10_H_19_O_11_^+^), 243.1074 (C_8_H_19_O_8_^+^)	-
46.	Trimethoxy-hydroxyflavone (salvigenin)	24.12	C_18_H_16_O_6_	−2.4	5.7	329.1028 (C_18_H_17_O_6_^+^)	**329.1027** (C_18_H_17_O_6_^+^), 314.0794 (C_17_H_14_O_6_^+^), 296.0688 (C_17_H_13_O_5_^+^)	-
47.	Pentacyclic terpenoid derivative (pentahydroxy-oleanen)	27.86	C_29_H_48_O_5_	0.3	7.3	477.3575 (C_29_H_48_O_5_^+^)	459.3469 (C_29_H_47_O_4_^+^), 441.3363 (C_29_H_45_O_3_^+^), 431.3520 (C_28_H_47_O_3_^+^), **413.3414** (C_28_H_45_O_2_^+^), 395.3308 (C_28_H_43_O^+^)	-
48.	Unidentified	28.77	C_28_H_44_O_11_	−5.9	38.6	557.2956 (C_28_H_45_O_11_^+^)	**465.2483** (C_25_H_37_O_8_^+^)	-

* Corresponds to the formate adduct; ** Corresponds to the ammonium adduct; numbers in bold represent the base peak.

**Table 2:** The cytotoxicity of *Iphiona mucronata* extract on different cell lines.

	VERO	FaDu		HeLa		RKO	
	CC_50_	CC_50_	SI	CC_50_	SI	CC_50_	SI
*Iphiona mucronata* extract	100.5 ± 18.2	101.7 ± 17.5	0.99	91.6 ± 18.5	1.1	54.5 ± 6.8	1.84

CC_50_—50% Cytotoxic concentration (µg/mL), Mean ± SD; concentration decreasing cellular viability by 50%; SI—Selectivity Index (SI = CC_50_VERO/CC_50_Cancer); Cell lines: VERO (ECACC, No. 84113001, kidney of a normal adult African Green monkey), FaDu (ATCC, HTB-43, human hypopharyngeal squamous cell carcinoma), HeLa (ECACC No. 93021013, human cervical adenocarcinoma), RKO (ATCC No. CRL-2577, human colon carcinoma).

## Data Availability

Not applicable.

## Supplementary Materials

The following supporting information can be downloaded at: https://www.mdpi.com/article/10.3390/molecules27217529/s1, Figures S1–S25.

## Abbreviations

Ag: aglycone; CQA, caffeoylquinic acid; FQA, feruloylquinic acid; GUAI, guaiano-lide; HRMS, high-resolution mass spectrometry; MEM, Minimum Essential Medium; DMEM, Dulbecco’s Modified Eagle Medium; PBS, phosphate buffered saline; FBS, fetal bovine serum; MTT, 3-[4,5-dimethylthiazole-2-yl]-2,5-diphenyltetrazolium bromide; SDS, sodium dodecyl-sulphate.
