# Supplementary material for: Iphiona mucronata (Forssk.) Asch. & Schweinf. A Comprehensive Phytochemical Study via UPLC-Q-TOF-MS in the Context of the Embryo- and Cytotoxicity Profiles
Source: Molecules. 2022 Nov 3;27(21):7529. doi: 10.3390/molecules27217529 (PMC9656354; doi:10.3390/molecules27217529)
Supplement: Supplementary file 1 [file molecules-27-07529-s001.zip › molecules-1985568-supplementary.pdf]

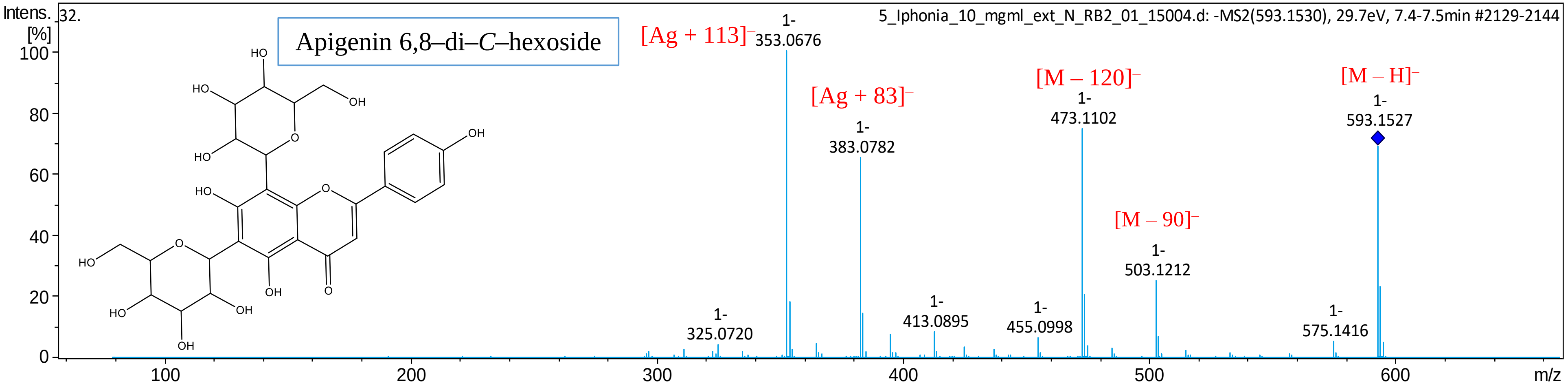

Supplementary Figure S1. MS/MS spectrum of peak **13** in the negative ion mode

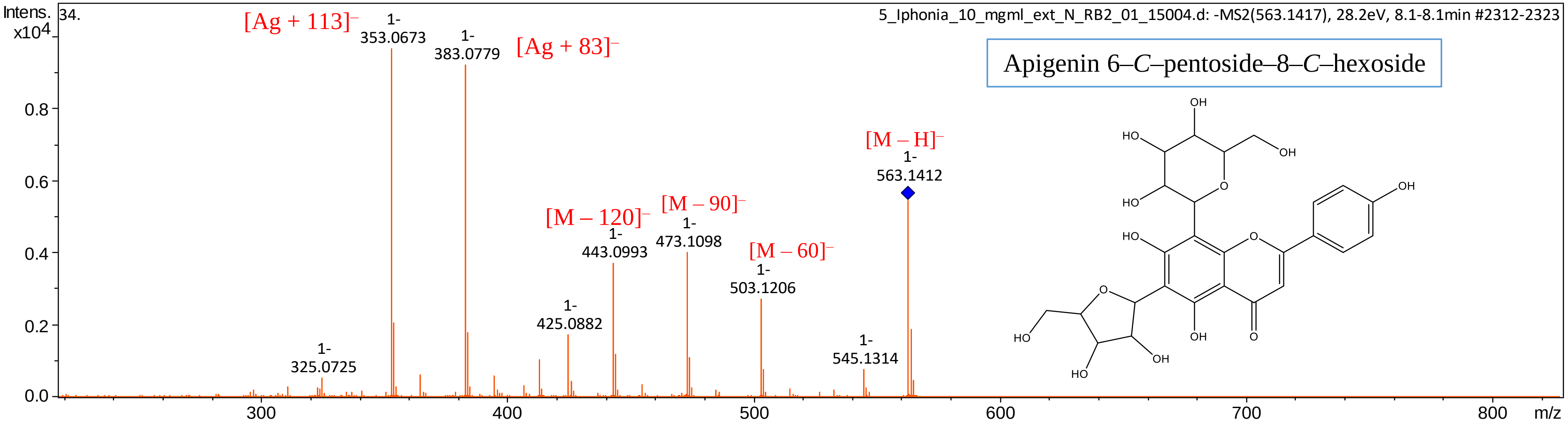

Supplementary Figure S2. MS/MS spectrum of peak **15** in the negative ion mode

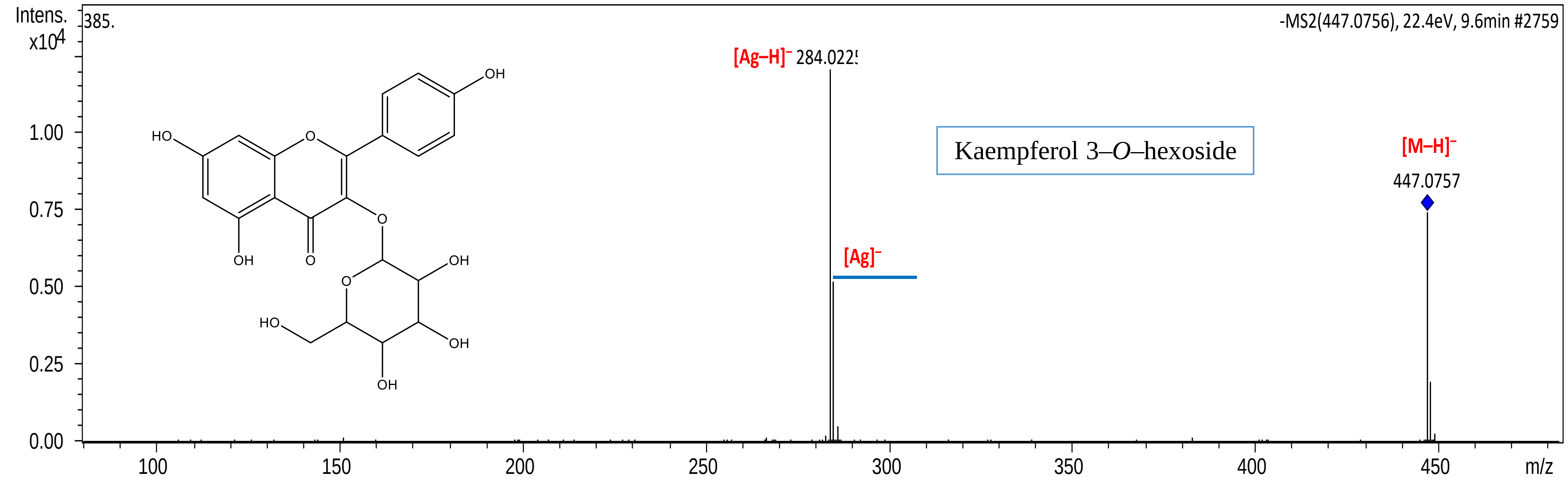

Supplementary Figure S3. MS/MS spectrum of peak **19** in the negative ion mode

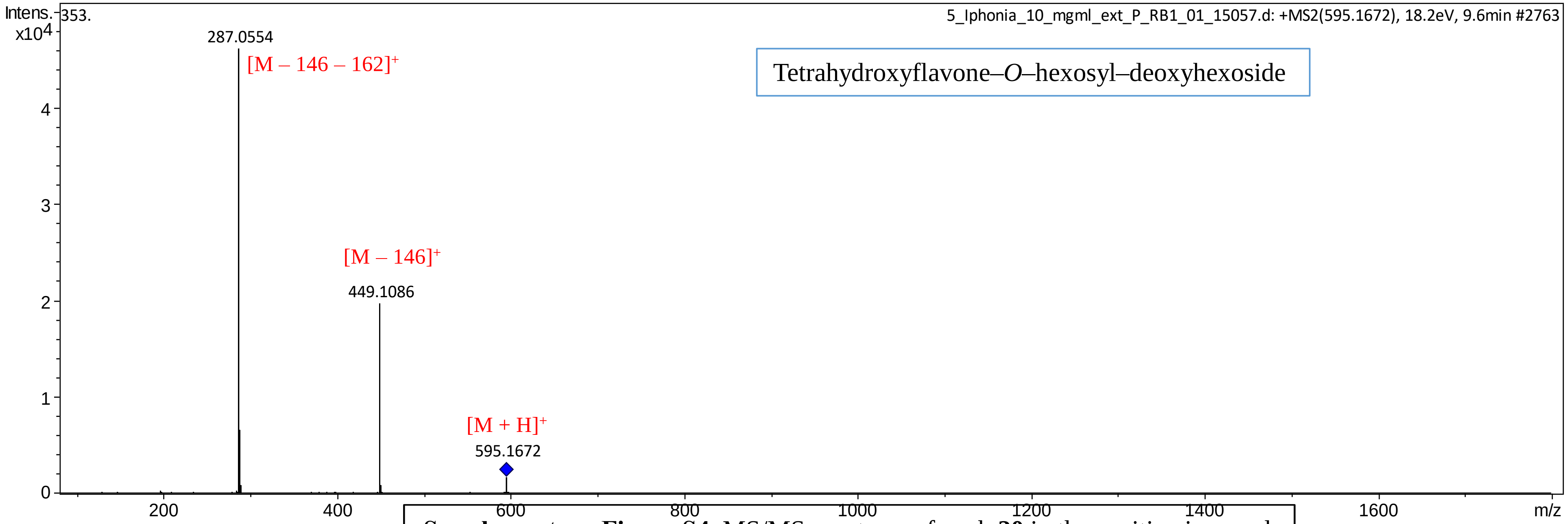

Supplementary Figure S4. MS/MS spectrum of peak **20** in the positive ion mode

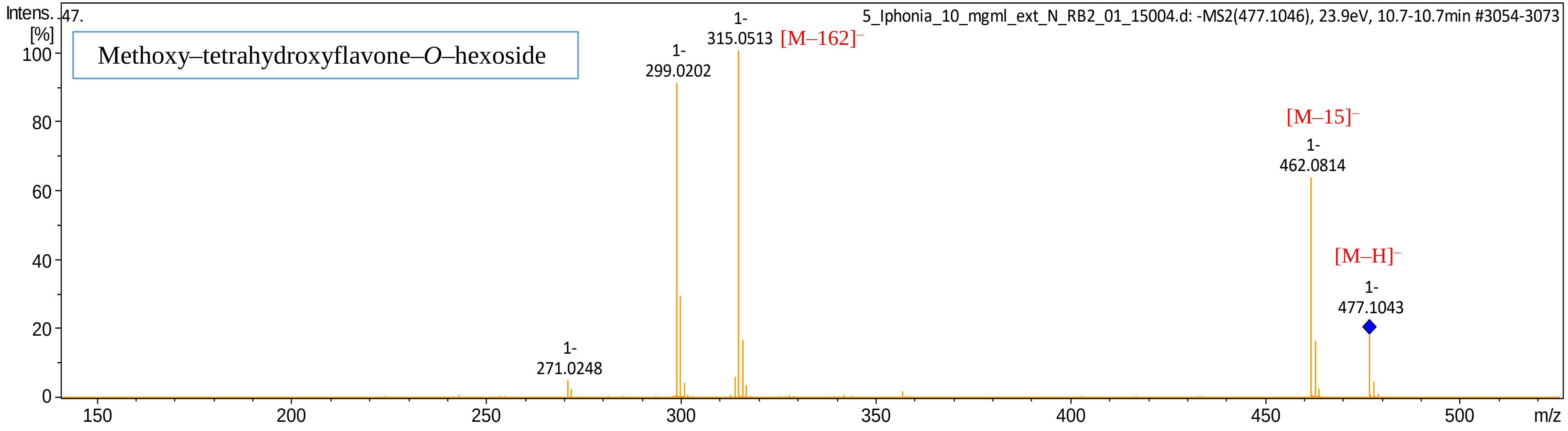

Supplementary Figure S5. MS/MS spectrum of peak **22** in the negative ion mode

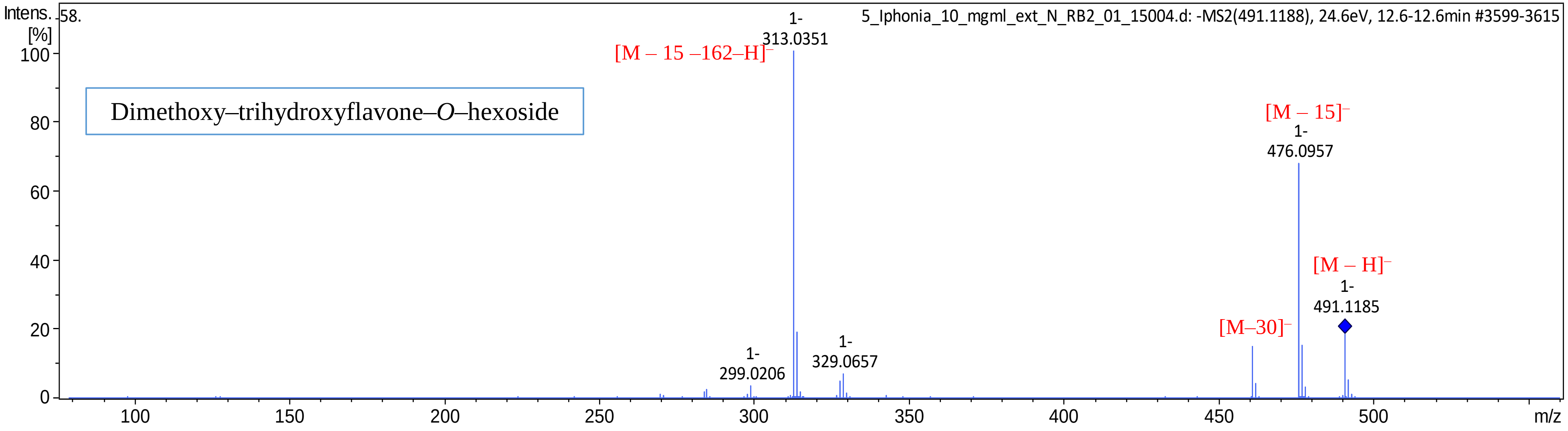

Supplementary Figure S6. MS/MS spectrum of peak **30** in the negative ion mode

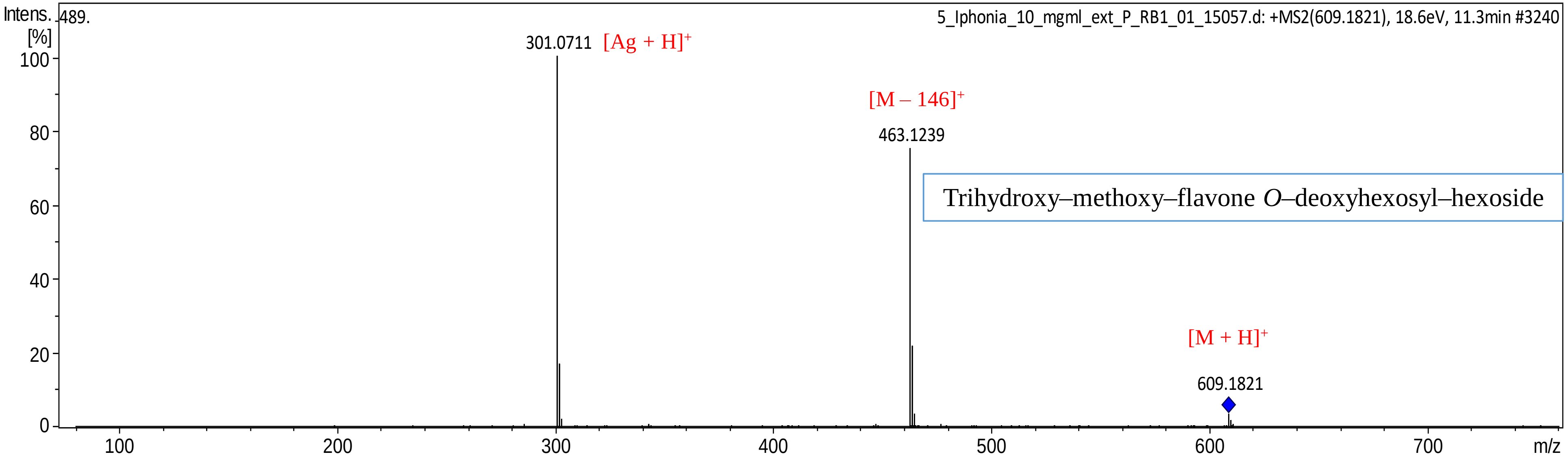

Supplementary Figure S7. MS/MS spectrum of peak **24** in the positive ion mode

Hydroxycinnamic acids

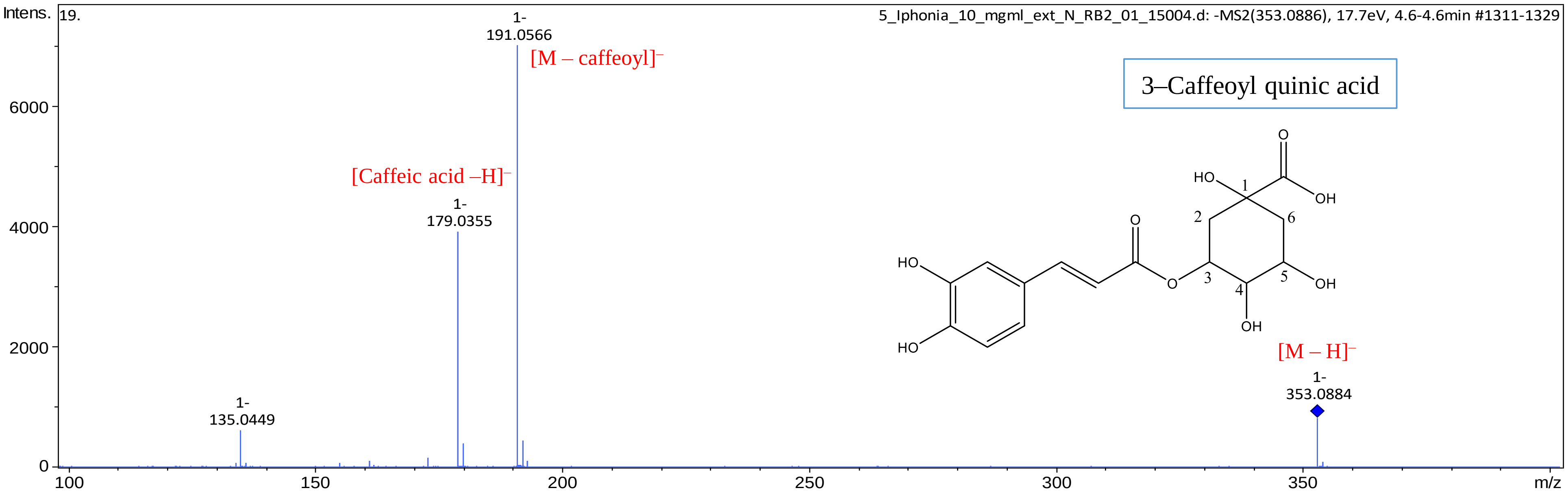

Supplementary Figure S8. MS/MS spectrum of peak **6** in the negative ion mode

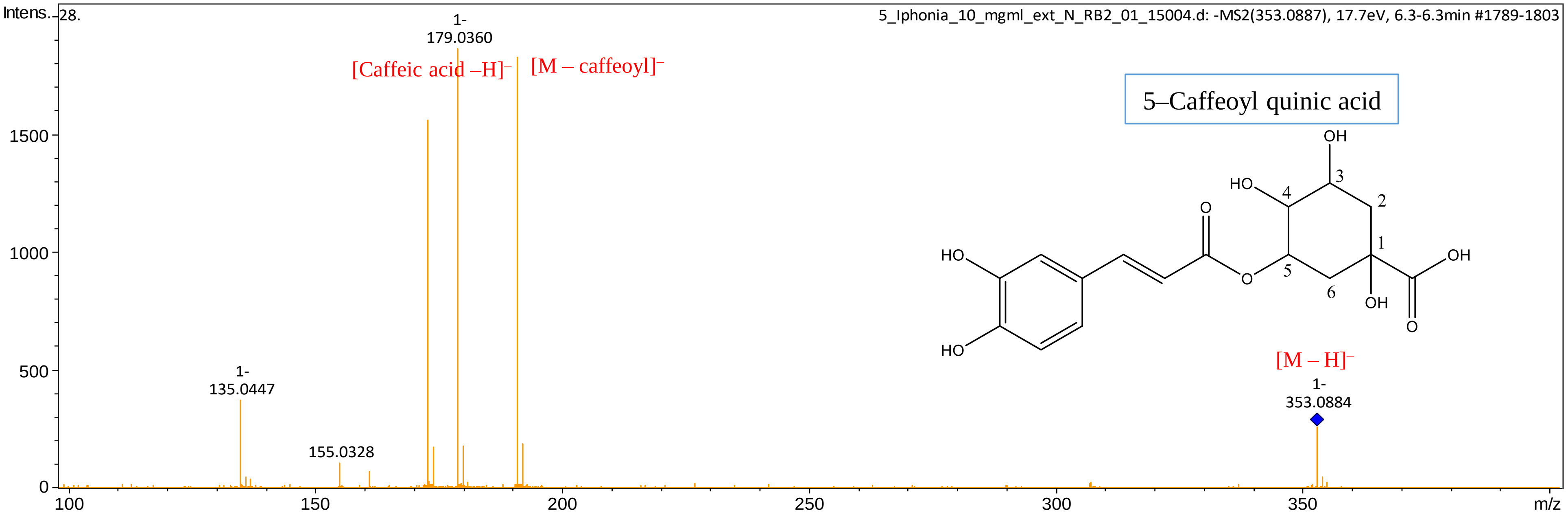

Supplementary Figure S9. MS/MS spectrum of peak **9** in the negative ion mode

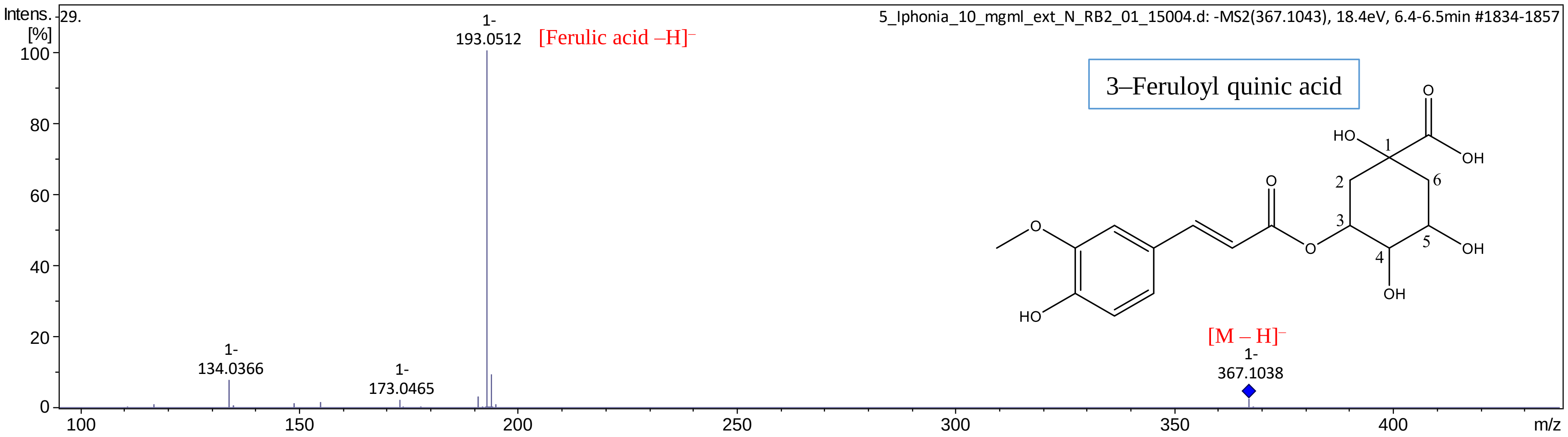

Supplementary Figure S10. MS/MS spectrum of peak **10** in the negative ion mode

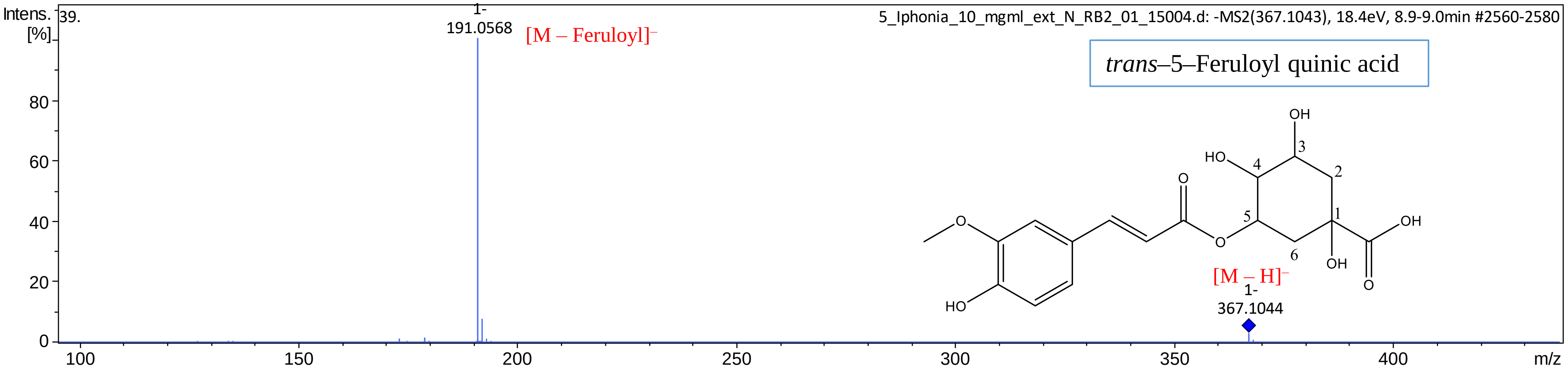

Supplementary Figure S11. MS/MS spectrum of peak **14** in the negative ion mode

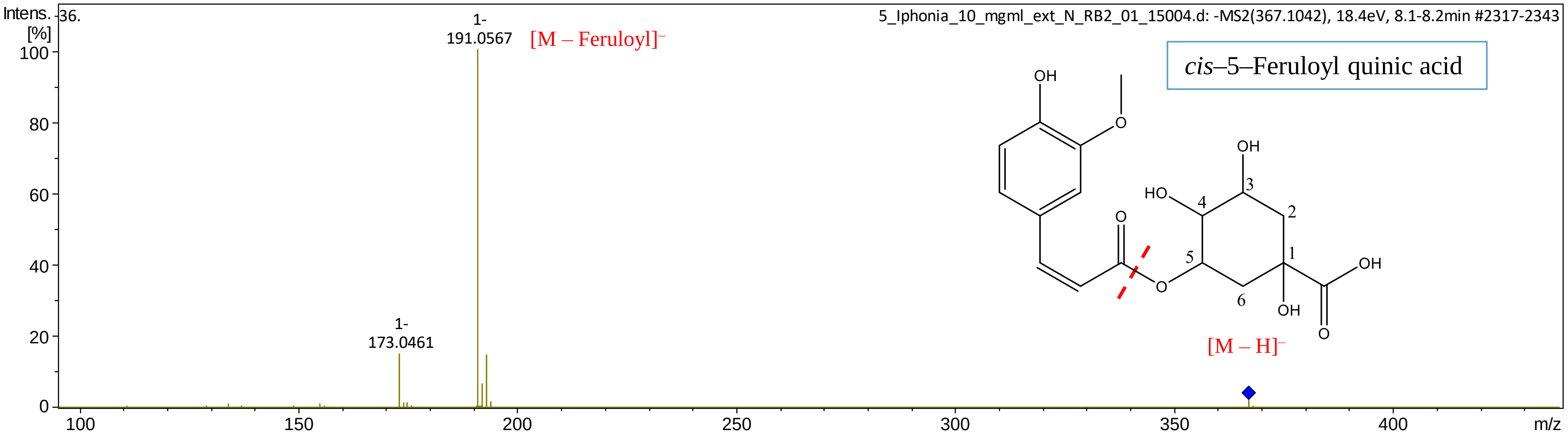

Supplementary Figure S12. MS/MS spectrum of peak **16** in the negative ion mode

Sesquiterpene lactones (Guaianolides)

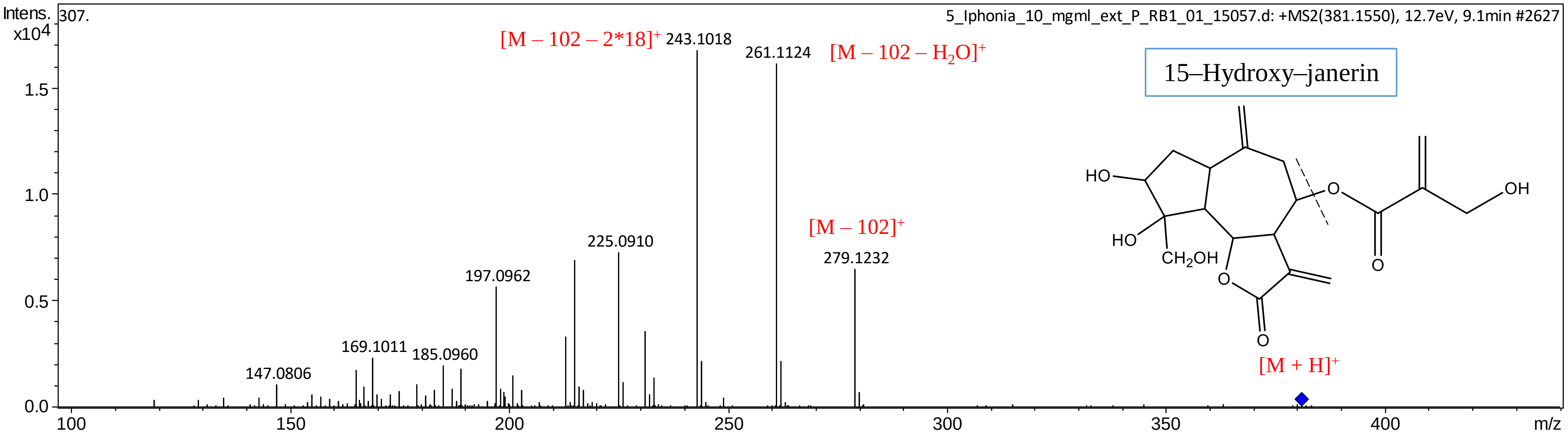

Supplementary Figure S13. MS/MS spectrum of peak **17** in the positive ion mode

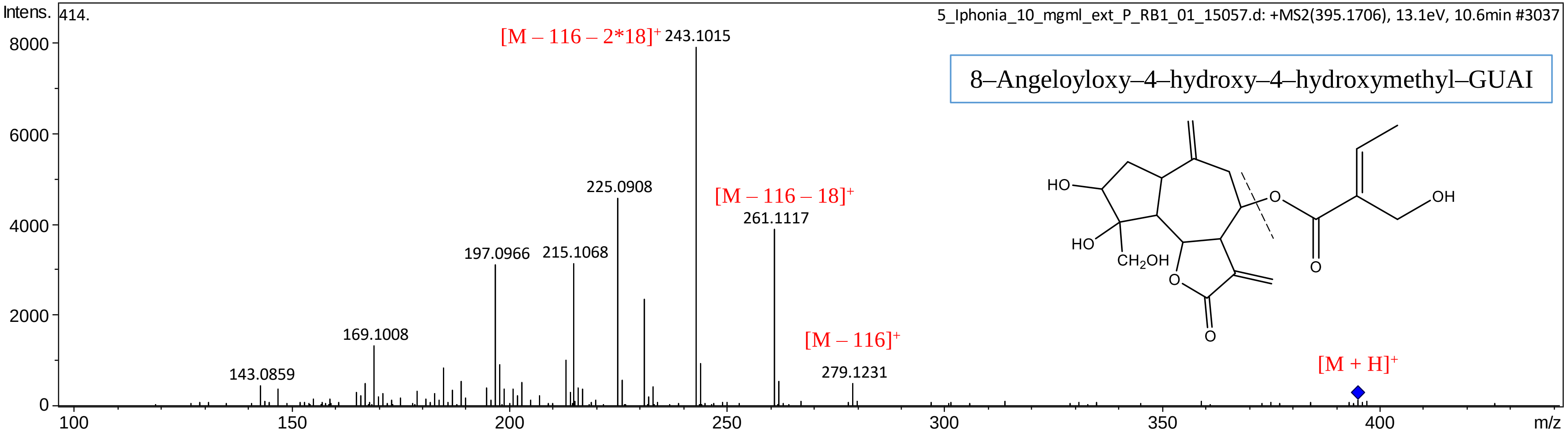

Supplementary Figure S14. MS/MS spectrum of peak **21** in the positive ion mode

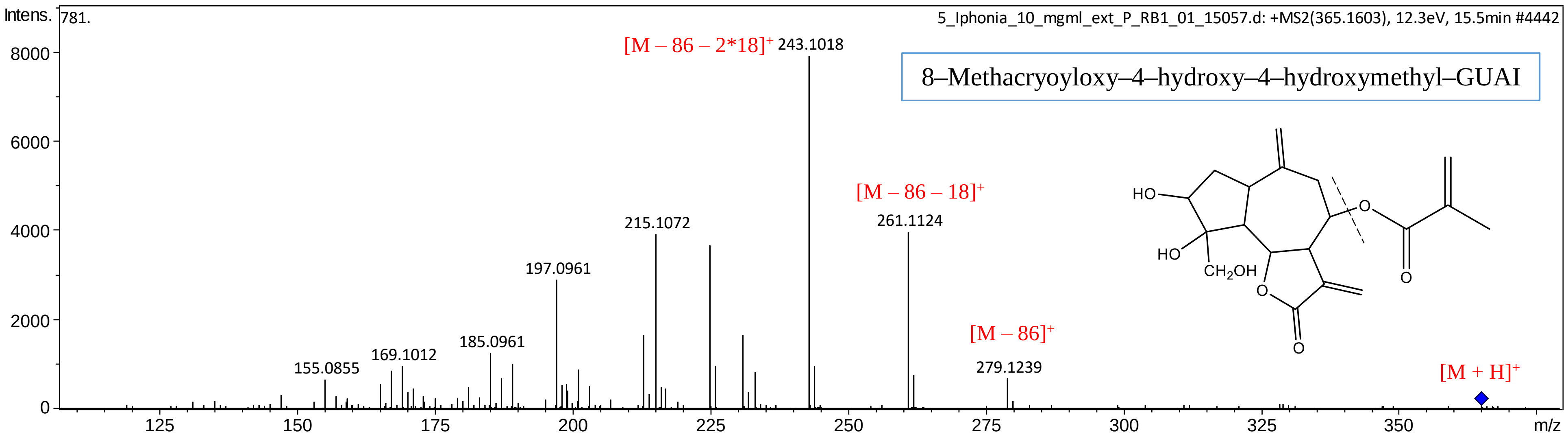

Supplementary Figure S15. MS/MS spectrum of peak **38** in the positive ion mode

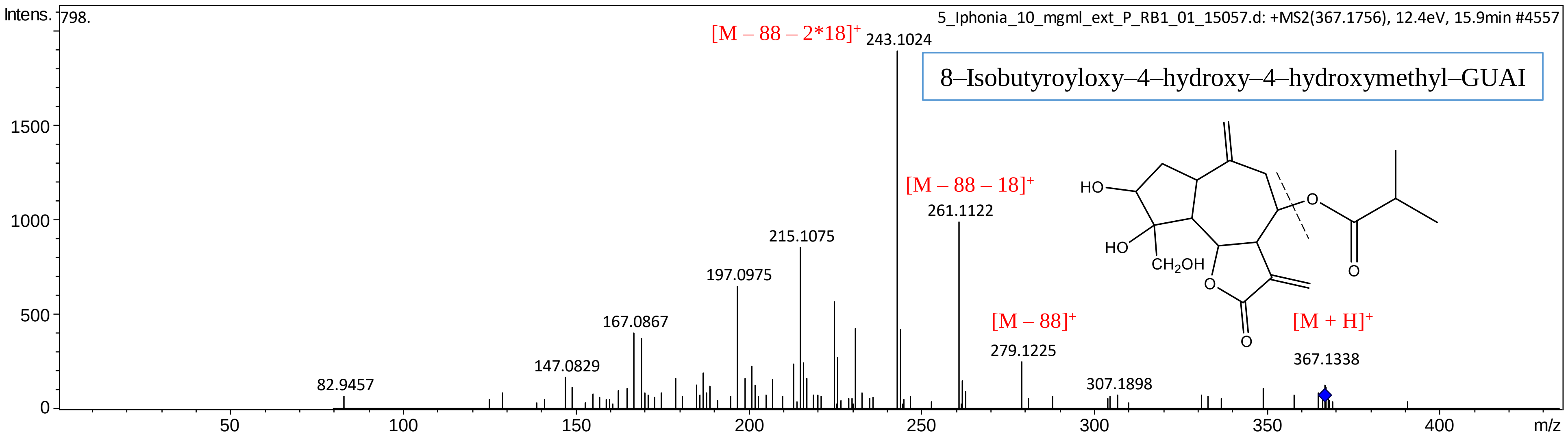

Supplementary Figure S16. MS/MS spectrum of peak **39** in the positive ion mode

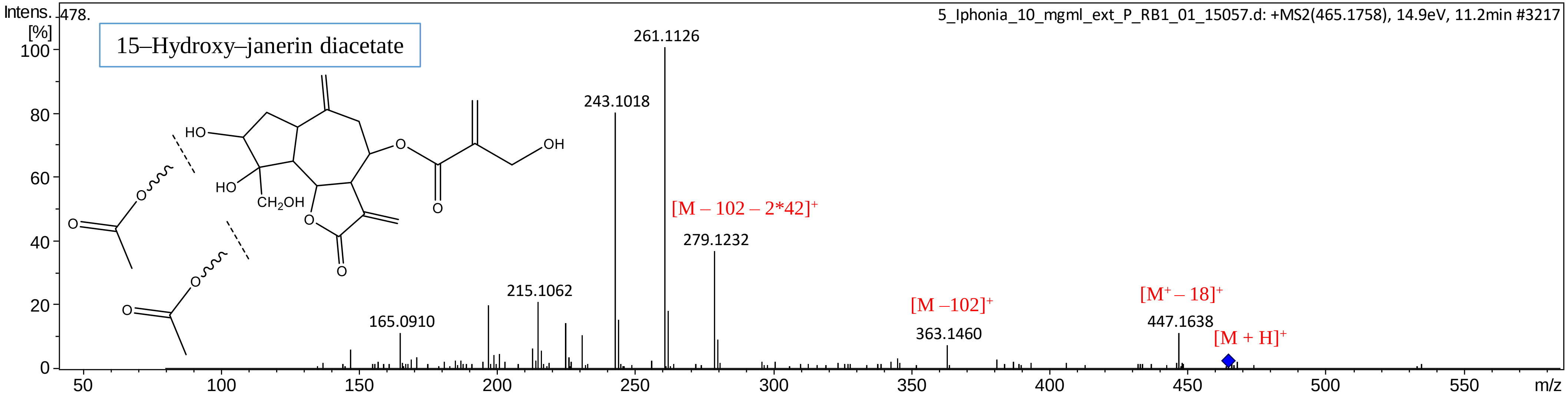

Supplementary Figure S17. MS/MS spectrum of peak **23** in the positive ion mode

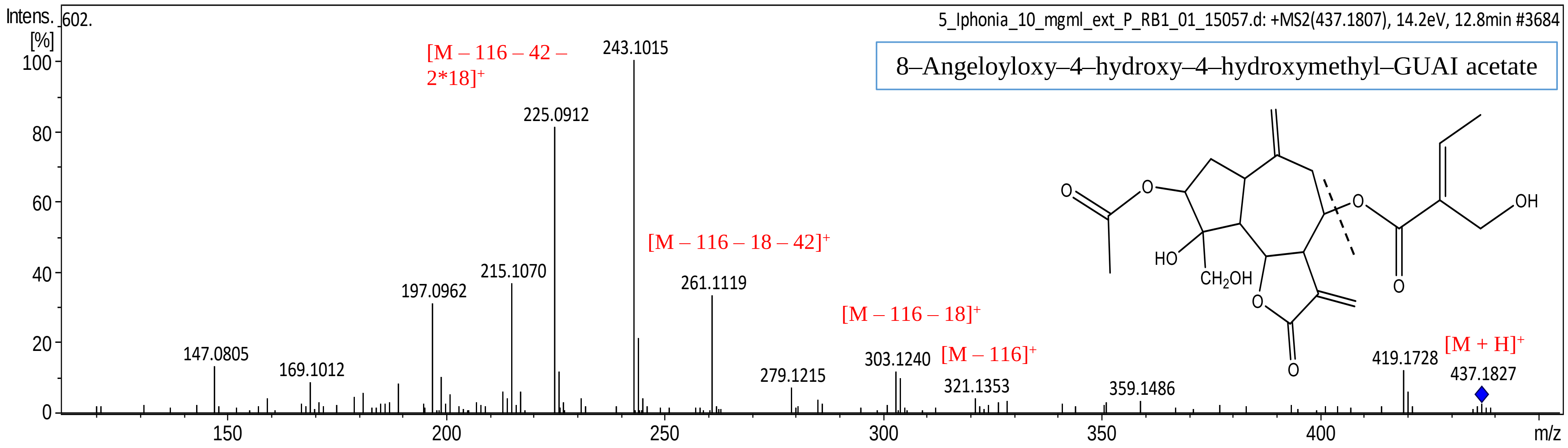

Supplementary Figure S18. MS/MS spectrum of peak **31** in the positive ion mode

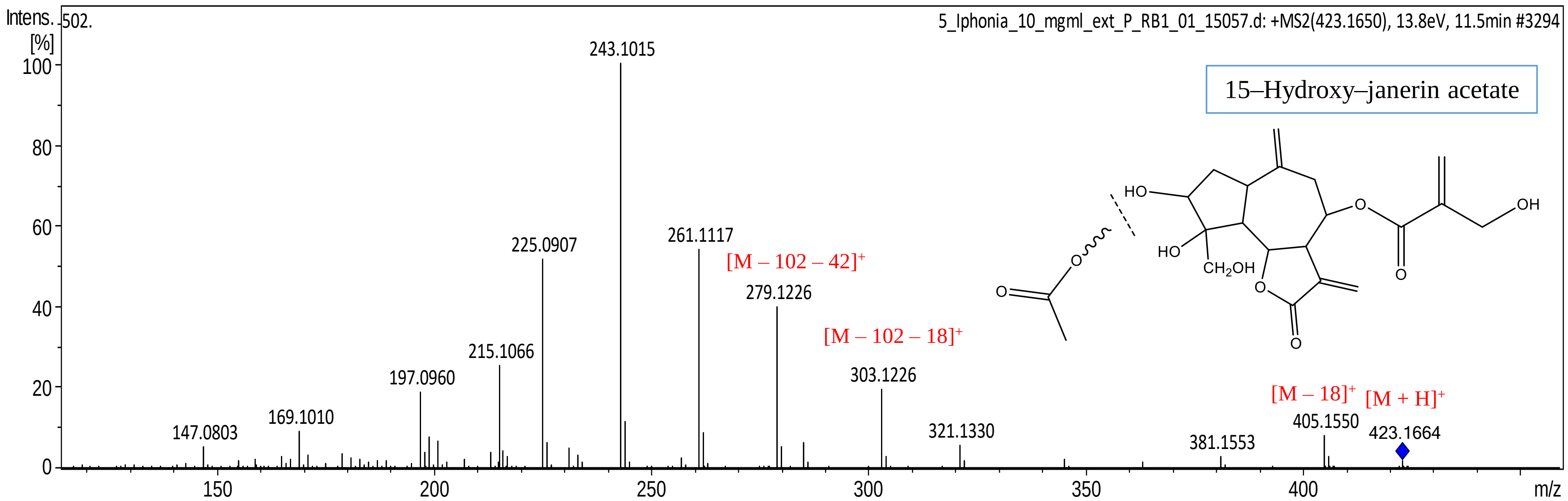

Supplementary Figure S19. MS/MS spectrum of peak **25/27** in the positive ion mode

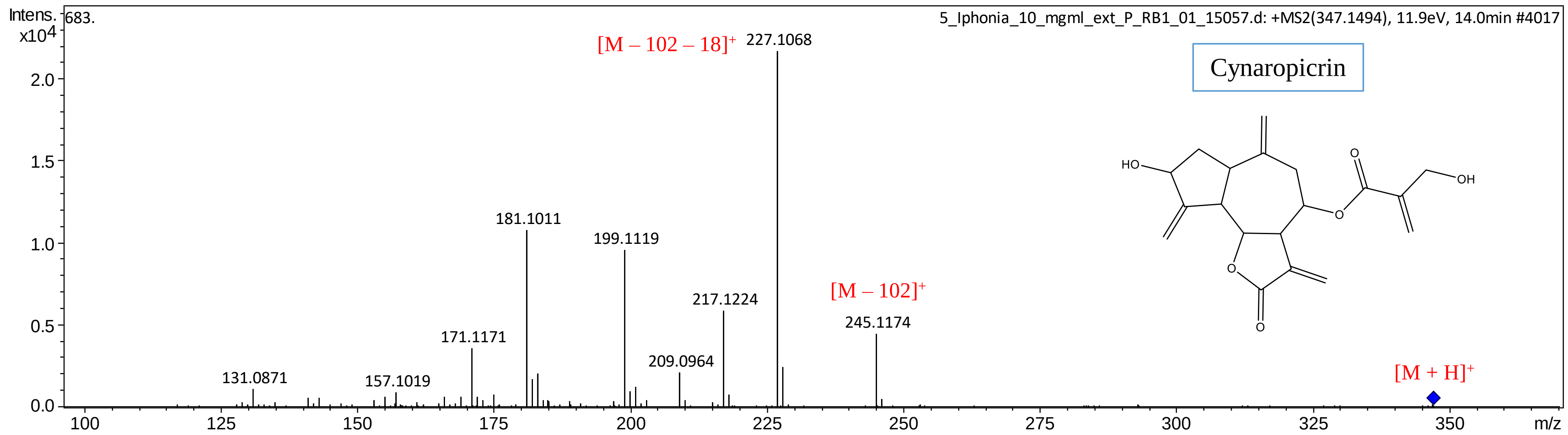

Supplementary Figure S20. MS/MS spectrum of peak **34** in the positive ion mode

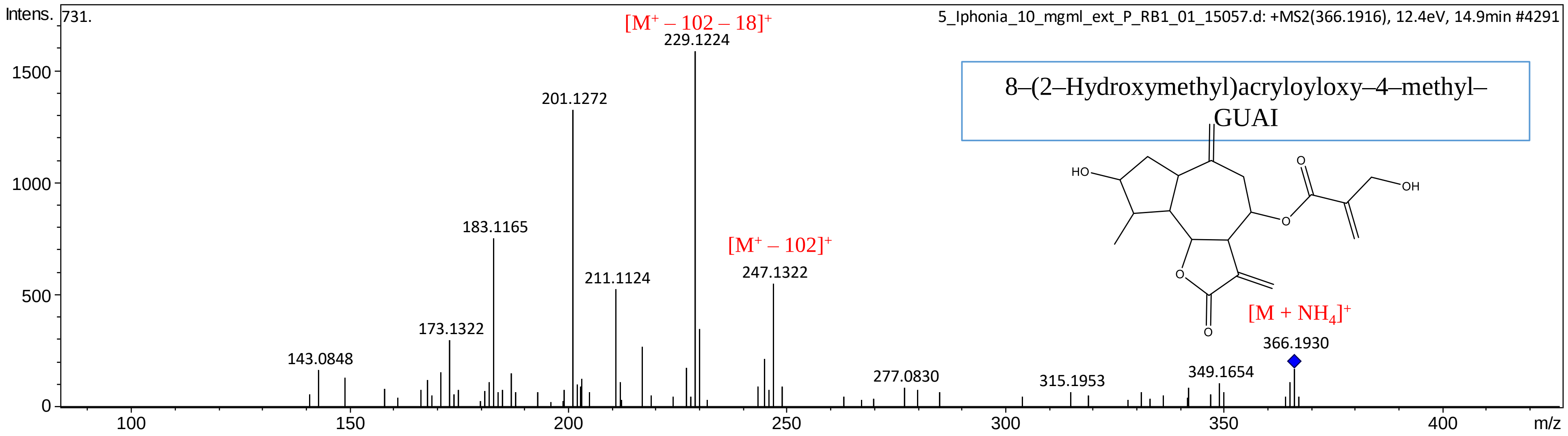

Supplementary Figure S21. MS/MS spectrum of peak **35** in the positive ion mode

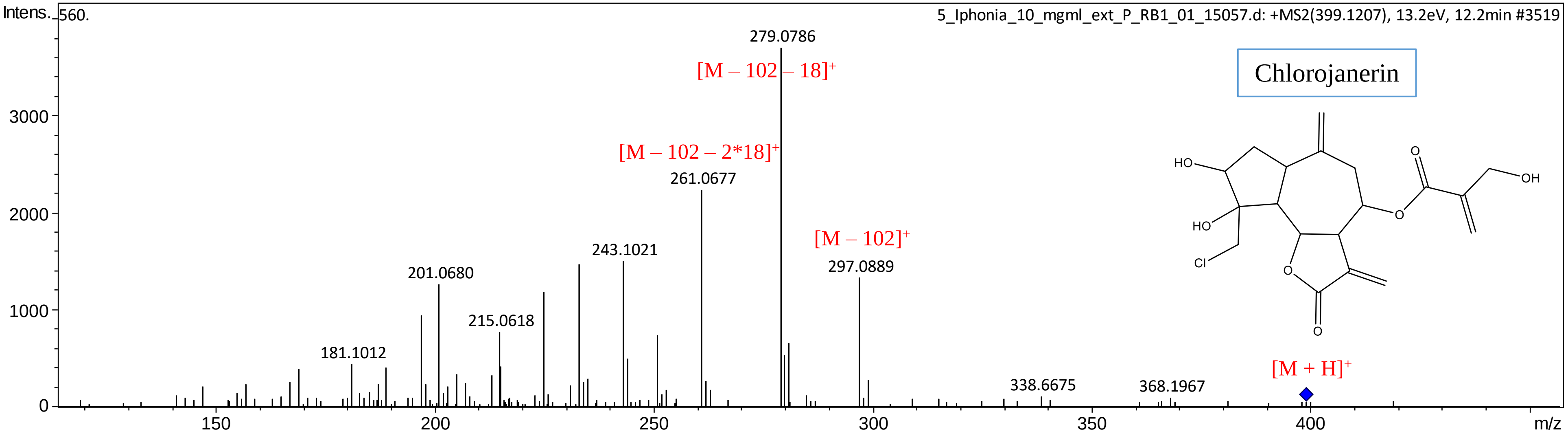

Supplementary Figure S22. MS/MS spectrum of peak **29** in the positive ion mode

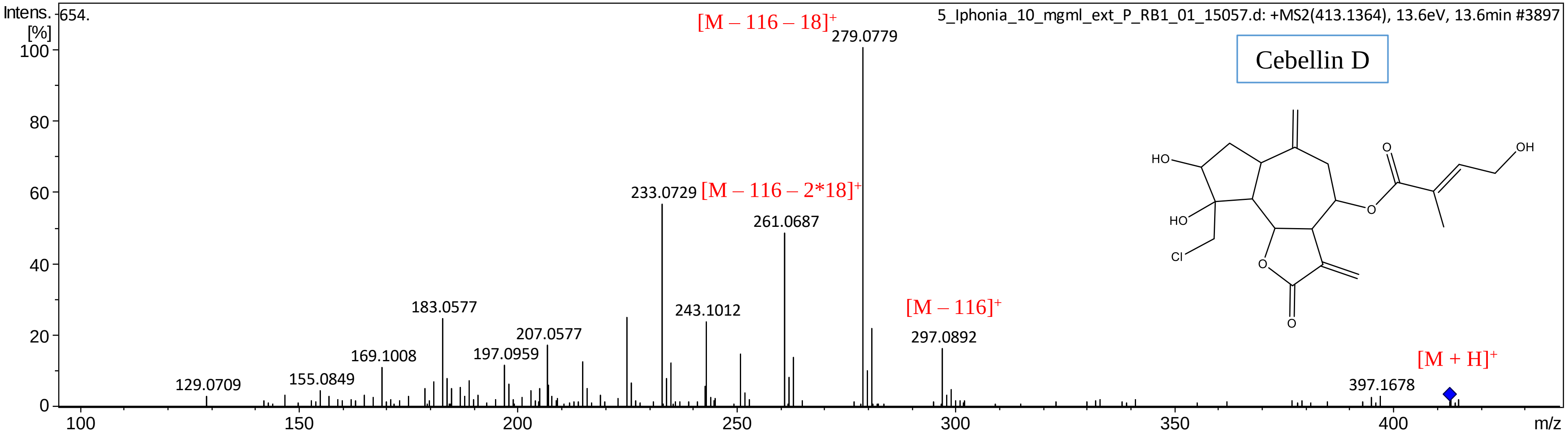

Supplementary Figure S23. MS/MS spectrum of peak **33** in the positive ion mode

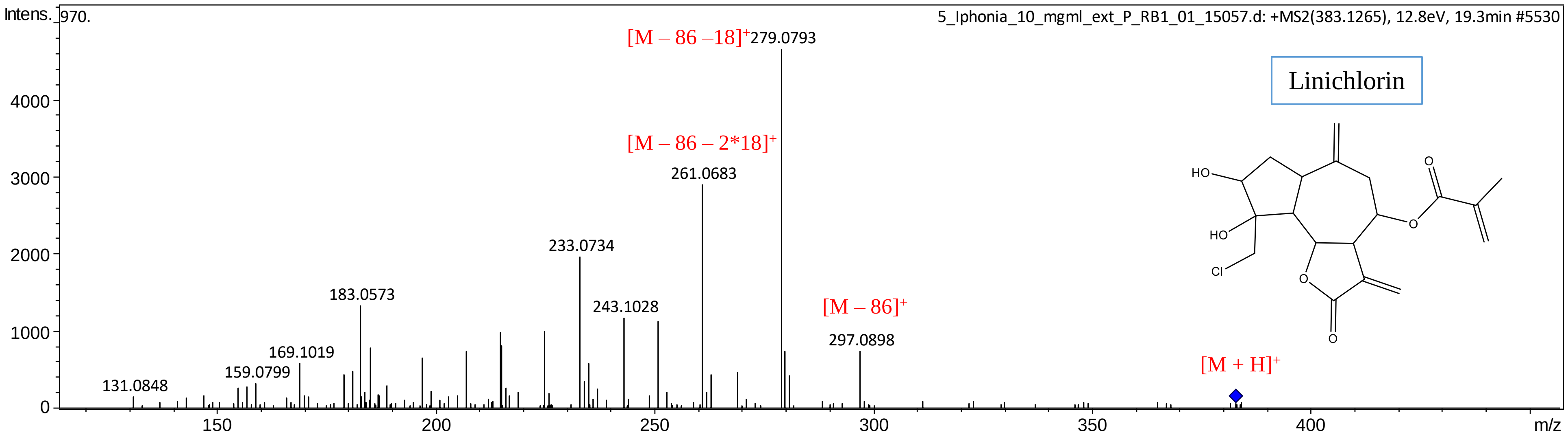

Supplementary Figure S24. MS/MS spectrum of peak **43** in the positive ion mode

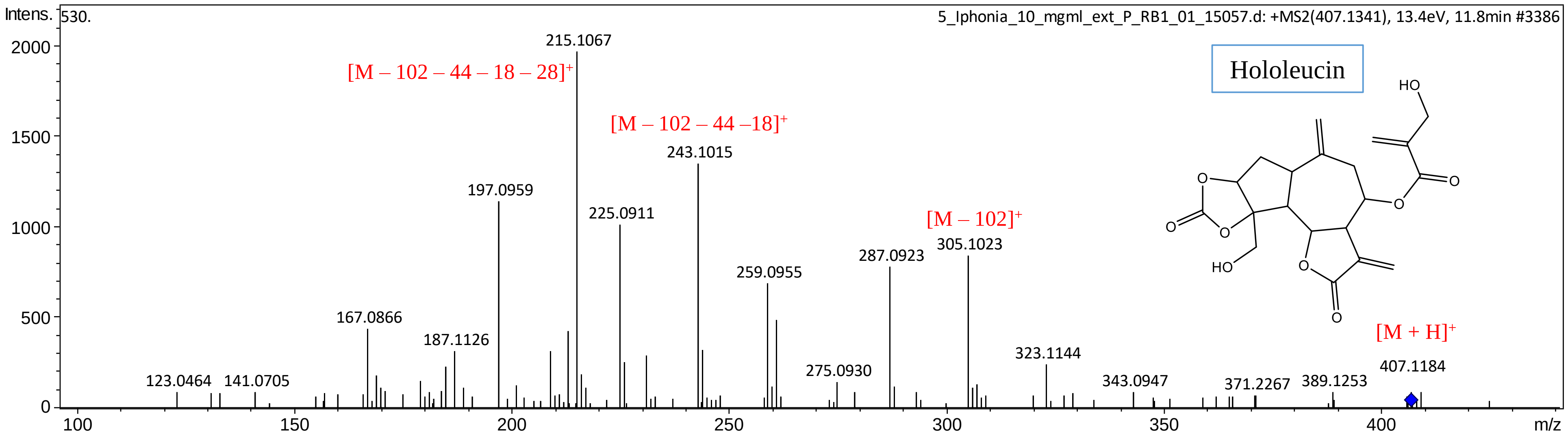

Supplementary Figure S25. MS/MS spectrum of peak **26** in the positive ion mode
